# Prevalence, host range, and characterization of multiple Palo verde broom emaravirus genomes and eriophyid mites from *Parkinsonia* spp. in Arizona

**DOI:** 10.1016/j.virusres.2025.199643

**Published:** 2025-10-16

**Authors:** Raphael O. Adegbola, Dinusha C. Maheepala, Ursula K. Schuch, Judith K. Brown

**Affiliations:** School of Plant Science, The University of Arizona, Tucson, AZ 85719, United States

**Keywords:** Emaravirus, Eriophyid mite vector, Palo verde tree, Witches’ broom

## Abstract

The palo verde tree is native to the Sonoran Desert and consists of multiple species classified in the genus *Parkinsonia*, family, Fabaceae. Palo verde broom virus (PVBV), *Fimoviridae, Emaravirus,* is the suspect causal agent of witches’ broom disease of blue palo verde, *P. florida*. Here, PVBV was detected in four palo verde species and two hybrids by reverse transcription polymerase chain reaction (RT-PCR) amplification of a 679-base pair (bp) fragment of RNA3, which encodes the nucleocapsid gene (NP). The prevalence of witches’ broom symptoms among the different *Parkinsonia* species (*n* = 70), collected from naturally-occurring, nursery- or urban landscape trees was 54 %. Within-species PVBV infection spanned 50–100 % and 81 % across four species and two hybrids combined. The PVBV genome segments RNAs 1–5 were *de novo* and reference based-assembled from Illumina® RNAseq reads obtained from total RNA isolated from PVBV-positive trees. Pairwise nucleotide identity and amino acid identity for 29 field isolates and GenBank reference PVBV RNA1–5 segments/predicted proteins was 73–100 % and 68–100 %, respectively. Phylogenetic analysis of concatenated RNA1–5 segments resolved four sister clades with no basis in host range among the four palo verde species or hybrids. Five predicted recombinants were identified with breakpoints in either tfhe RNA1 or RNA5 genomic segment. Consistent recovery of PVBV full-length genomes from four *Parkinsonia* spp. and two hybrids indicated that additional *Parkinsonia* species and hybrids besides blue palo verde, the only previously reported host, harbored PVBV. Previous studies have linked emaravirus transmission with *Eriophyidae* mite vectors. Here, the palo verde mite *Aculus cercidii* Keifer (*Eriophyidae*) (1965) counts ranged from eight to >1000 per tree. Prolific or minimally-detectable colonization of PVBV-infected trees by *A. cercidii*, together with consistent detection of PVBV in symptomatic and asymptomatic trees implicate the palo verde mite as the vector of and PVBV as the causal agent of witches’ broom disease.

## Research highlights

• Characterization and phylogenetic relationships of twenty-nine palo verde broom virus isolates.
• First evidence that PVBV infects four *Parkinsonia* spp. and two *Parkinsonia* x hybrids.
• Genomic variability, manifest as divergent RNA segments or genome copies.
• Ecological insights into the palo verde host-emaravirus-eriophyid mite vector pathosystem.

## 1 Introduction

The palo verde tree (*Parkinsonia* spp.) is the common name for several species of drought- and heat-tolerant leguminous trees that are native to and widely distributed in the Sonoran Desert, which encompasses portions of the southwestern U.S. and northwestern Mexico (Carter, 1974; Schuch and Kelly, 2012; Spurrier and Smith, 2006). The *Parkinsonia* genus contains species that range in size from large shrubs to medium-sized trees, and are the most important nurse plant that provides shade and protection for the Saguaro cactus (*Carnegiea gigantea*; (Drezner, 2010)). The pods are consumed as a vitamin-rich food by people native to the Sonoran Desert (Sutton, 1987) and the trees provide food and shelter for native wildlife. The palo verde tree is also valued as a landscape tree to further environmental conservation (Bainbridge and Virginia, 1989; Bowers and Turner, 2001; 2002; Schuch and Kelly 2012). The best-known species of palo verde are the blue palo verde (*Parkinsonia florida* Benth. ex A. Gray), Desert Museum palo verde ((*P*. x ‘Desert Museum’ syn. *P. aculeata* x *P. microphylla*) x *P. florida*), foothills or little leaf palo verde (*P. microphylla* Torr.), Mexican palo verde (*P. aculeata* L.), Sonoran palo brea (*Parkinsonia praecox* subsp. *praecox*), and Sonoran palo verde (*P*. x *sonorae* syn. *P. microphylla* x *P. praecox*) (Bowers and Turner, 2001; Schuch and Kelly, 2012). The blue and foothills palo verde are designated as the official state trees of Arizona.

Several arthropod pests and diseases of palo verde trees have been reported, including witches’ broom (Bainbridge and Virginia, 1989; Schuch and Kelly, 2012), for which the etiology was unknown until recently. Characteristic witches’ broom disease symptoms in palo verde trees consist of shoot proliferation, dense twig growth, prolific development of suckers on and at the base of the primary trunk, branches, shortened internodes, defoliation, stem die-back, necrosis, and decline were first noted by (Dimmit, 1987). Characteristically, witches’ broom symptoms have been associated with phytoplasma etiology (Bové et al., 2000; Pardo et al., 2023; Quaglino et al., 2015; Zamharir et al., 2011), however, a number of emaravirus-eriophyid mite complexes also can cause foliar and floral deformation reminiscent of phytoplasma-incited diseases of herbaceous and woody plants (Di Bello et al., 2015; Elbeaino and Digiaro, 2021; Hoat et al., 2017; Keifer, 1982; Oldfield and Proeseler, 1996; Seo et al., 2017). During the preceding fifteen years, witches’ broom disease symptoms have become increasingly widespread in palo verde trees in nursery and urban landscape palo verde trees throughout the Sonoran Desert (Schuch et al., 2022; Schuch and Kelly, 2012). This prompted an investigation into causality of the disease that has led to the discovery of an emaravirus, named palo verde broom virus (PVBV), taxonomically classified as *Emaravirus parkinsonae* associated with blue palo verde trees exhibiting witches’ broom symptoms (Ilyas et al., 2018). The International Committee on Taxonomy of Viruses (ICTV) Fimoviridae Study Group has recognized PVBV as a new emaravirus species, based on the *Emaravirus* species cutoff of >20 % divergence among the amino acid sequences of at least two of the gene products of four core proteins (RNA1–4) (Digiaro et al., 2024).

Emaraviruses have quasi-spherical, enveloped virions of 80–150 nm in diameter and are transmitted by eriophyid mites (Digiaro et al., 2024; Elbeaino and Digiaro, 2021). Emaraviruses have a segmented, negative-sense, single-stranded RNA genome (-ssRNA) that consist of 4–10 segments ranging from 12.3–18.5 kb in size (Digiaro et al., 2024; Rehanek et al., 2022). Emaravirus genome segments are not capped or polyadenylated, however, they have a unique conserved, nearly perfect complementary 18–20 nt sequence located at the 5′- and 3′- termini (Digiaro et al., 2024; Elbeaino et al., 2009; Rehanek et al., 2022). The complementary strand (3′← 5′) genomic RNA of each of the genome segments encodes a single protein. The RNA1 encodes the RNA-dependent RNA polymerase (RdRp; ∼266 kDa), RNA2 encodes the glycoprotein (GP; 75 kDa), and the RNA3 and RNA4 encode the nucleocapsid protein (NP; 35 kDa), and RNA4 movement protein (MP; 42 kDa), respectively. Emaraviruses may have up to three additional RNA segments that encode a single protein each, referred to as RNA5–8 (Digiaro et al., 2024). The function(s) of most emaravirus-encoded proteins is unknown. However, the P5 protein encoded by RNA5 of pear chlorotic leaf spot-associated virus (PCLSaV), taxonomically classified as *Emaravirus pyri*, has been shown to suppress systemic RNA silencing and trigger the accumulation of reactive oxygen species (Ren et al., 2024), and P7 and P8, encoded by the HPWMoV RNA7 and 8, respectively, have been implicated as suppressors of host plant gene-silencing (Gupta et al., 2019, 2018).

The PVBV genome sequence from blue palo verde tree has been reported to comprise four RNA segments, RNAs 1–4, of approximately 7, 2, 1.4, and 1.5 kg bases (kb), respectively (RNA1, MF766025; RNA2, MF766030; RNA3, MF766035; and RNA4, MF766040) (Ilyas et al., 2018). The RNA1 genome segment encodes a predicted RNA-dependent RNA polymerase, while RNA2 encodes the envelope glycoprotein, and RNAs 3 and 4 encode the nucleocapsid and movement protein, respectively. At the genome sequence level, the PVBV RNA1–4 segments share their greatest pairwise nucleotide identity with high plains wheat mosaic virus (HPWMoV) (Stewart et al., 2013), at 60–65 % (Ilyas et al., 2018). Pairwise distance analysis of PVBV P1–4 predicted amino acid sequences with other well-characterized emaraviruses has led to the recognition of PVBV as a distinct emaravirus species (Kuhn et al., 2021). Recently, a fifth PVBV RNA segment has been identified from blue palo verde trees, together with the four RNAs reported previously. All five of the RNAs were shown to contain the canonical emaravirus-like 3′- and 5′- AGCAGTAGTGATCTCCC sequence indicating that the PVBV genome consists of five RNA segments. Further, PVBV-infected blue palo verde trees have been shown to contain elevated levels of phytohormones analogous to those associated with defense responses in plants infected with viruses and other plant pathogens, and small-interfering RNAs (siRNAs) that corresponded to the five PVBV RNA genome segments, indicative of post-transcriptional gene silencing (Adegbola et al., 2025).

The eriophyid mite, *Aculus cercidii* Keifer (1965) (aka palo verde mite) was described from infested *P. microphylla* trees, or foothills palo verde, in Pima County, Arizona (Keifer, 1982) as a vagrant mite. However, neither presence of a significant mite population size or witches’ broom disease symptoms were documented in this first report. Preliminary observations have shown that *A. cercidii* frequently colonizes broom-symptomatic nursery-grown and urban-planted palo verde trees in southern Arizona. In a previous study *A. cercidi* was shown to reach high population densities on symptomatic blue palo verde trees, compared to low-level to undetectable infestations on asymptomatic trees (Ilyas et al., 2018, Schuch and Kelly, 2012). Finally, all well-characterized emaraviruses that colonize herbaceous and woody hosts have been associated with a host-specific eriophyid mite species (De Lillo et al., 2018; Druciarek and Tzanetakis, 2025; Oldfield and Proeseler, 1996). These collective observations have led to the hypothesis that *A. cercidii* is the eriophyid mite vector of PVBV.

The objectives of this study were to determine PVBV-disease prevalence in relation to broom symptomatology in palo verde trees using reverse transcription-polymerase chain reaction (RT-PCR) amplification of RNA isolated from locally-occurring palo verde species and hybrids, determine the complete PVBV genome sequence of multiple isolates from different host species, and determine the extent of genome variability among PVBV-isolates. The palo verde trees included in the study consisted of the following four species and two hybrids, the blue palo verde, the foothills or little leaf palo verde, the Mexican and Sonoran palo verde, and the Desert Museum and *P*. x hybrids. Multiple PVBV genome sequences were determined, and characterized with respect to recombination and PVBV-intraspecific genome variability. Finally, given that emravirus transmission has been linked to host-specific eriophyid mite vectors (Mielke-Ehret and Mühlbach, 2012; Rehanek et al., 2022; Seifers et al., 1997) the presence or absence of mites was recorded, and when present, the number of mites was tabulated for selected PVBV-symptomatic and -asymptomatic trees from which the PVBV genome was characterized.

## 2 Materials and methods

### 2.1 Symptom surveys, plant collection, and eriophyid mite counts

Surveys of palo verde tree species with and without PVBV disease symptoms were carried out from July 2019 to November 2023. Mature *Parkinsonia* species and hybrid trees growing in urban landscapes in the Phoenix and Tucson metropolitan areas in southern Arizona were sampled and scored for presence or absence of characteristic symptoms of witches’ broom disease. Leaves from symptomatic small branches were collected by removing the terminal shoots from trees with pruners and placing branches immediately into a plastic bag and sealed. The samples were placed in an ice chest with icepacks, transported to the University of Arizona (Tucson, AZ) laboratory, and held at 4 °C until eriophyid mites (selected samples) were counted or placed immediately at −80 °C freezer (all samples), respectively.

### 2.2 Palo verde mite collection, identification, and tabulation on palo verde leaves

*Mite collections and morphological identification.* To tabulate the eriophyid mite *A. cercidi* colonizing individual trees, the number of mites visible on the shoot tips, including abaxial leaflet surfaces, buds, petioles, and branches of the collected shoots were counted using a Nikon SMZ80 stereo microscope (Nikon Instruments Inc., Melville, NY). Eriophyid mites were collected from the abaxial side of nodes and stem internodes of the leaflets removed from field-infested palo verde trees using an eye-lash brush. For identification, the mite-infested leaves were sealed in a plastic bag and shipped live in an insulated foam box ("8 × 6 × 7″) containing frozen gel packs to the USDA-ARS Systematic Entomology Laboratory, Beltsville Agriculture Research Station, Maryland.

Scanning electron microscope (SEM) observations were made with a TM 3030Plus tabletop SEM (Hitachi High-Tech America, Inc., Schaumburg, IL) equipped with a Deben cooler system (Angstrom Scientific Inc., Ramsey, NJ). Eriophyid specimens in situ on plant material were attached to 32 mm aluminum specimen holders with carbon sticky pads. The samples were cooled to −25 °C. An accelerating voltage of 5 kV was used to view the specimens and their interaction with the leaf substrata. Adult mites, immatures and eggs were observed and imaged. Imagery was compared with specimens in the Smithsonian National Mite Collection as well as the original description (Keifer, 1982).

### 2.3 Total RNA isolation

For RNA isolation, cohorts of five or ten eriophyid mites (previously identified as the palo verde mite) were collected from the leaflets of palo verde plants from a laboratory-maintained colony, and placed into a 2 ml screw-cap tube containing beads and DNA/RNA protection reagent (New England Biolabs, Ipswich, MA). Leaf samples were ground in liquid nitrogen with a pre-cooled mortar and pestle. Total RNA was isolated using Fruit-mate (Takara Bio USA, San Jose, CA)/TRIzol LS reagent (ThermoFisher Scientific, Waltham, MA), or using NEB Total RNA isolation kit (New England Biolabs, Ipswich, MA) according to the manufacturer’s instructions. The quantity and quality of total RNA were determined using a NanoDrop One spectrophotometer (ThermoFisher Scientific) and Qubit Fluorometer with the RNA HS assay kit (Invitrogen, Carlsbad, CA), respectively.

### 2.4 Reverse-transcriptase PCR amplification and confirmatory Sanger sequencing

The complementary-strand DNA (cDNA) synthesis reaction was carried out using the two-step SuperScript IV reverse transcription (RT) system (Invitrogen). First-strand synthesis was primed with random primers and a universal virus-specific primer, PVBV-3C (Table 1), corresponding to the terminal 14 ribonucleotides at the 3′-terminal end of viral RNA genome segments. The reverse transcription-polymerase chain reaction (RT-PCR) amplification was carried out using the primer pair, PVBV-RNA3_433F/1111R (Table 1), designed to amplify a 679-base pair (bp) fragment of the nucleocapsid (NP) gene. Reactions were carried out in a final volume of 25.0 µl, containing REDTaq® ReadyMix™ (Sigma-Aldrich, St. Louis, MO), 0.4 µM of each primer, 2.0 µl cDNA template, and molecular biology grade water. Cycling conditions were initial denaturation: 95 °C for 2 min followed by denaturation at 95 °C for 30 s, annealing at 59 °C for 30 s, and extension at 72 °C for 50 s, and a final extension of 72 °C for 10 min. The appropriate expected sized of the PCR product was confirmed by electrophoresis on a 1 % agarose gel in Tris-acetate-EDTA (TAE) buffer, pH 8.0 containing 1X GelRed (Biotium, Fremont, CA), and photographed using the Molecular Imager Gel Doc System (BioRad, Hercules, CA). Amplicons of the expected size were ligated into the pGEM®-T Easy plasmid vector (Promega Corp., Madison, WI) and transformed into chemically-competent *Escherichia coli* strain DH5α cells. Individual colonies containing plasmids bearing the expected size insert were identified by colony PCR amplification (Bergkessel and Guthrie, 2013). Plasmids containing the cloned inserts were subjected to bidirectional DNA (Sanger) sequencing (Eaton Bioscience; https://www.etonbio.com/sequencing/dna_sequencing_services.php).

**Table 1:** Primers designed to prime complementary strand DNA synthesis, amplification of partial-length and complete RNA segments of palo verde broom virus.

Primer name	Purpose	Primer sequence*	Amplicon size (bp)⁎⁎	Tm ( °C)⁎⁎⁎	Reference
PVBV-3C	cDNA synthesis	CTCAGCAGTAGTGTACTCCC	–	–	Zheng et al. 2016@
RNA3_433F	RNA3 fragment	AYTCTCCWGTCTTCTCTTCATCA	679	59	This study
RNA3_1111R		GTCCGTAGCATTGACTGTGA			
PVBV-R2–1F	RNA2 segment	AGTAGTGATCTCCCCAAAACAACC	2114	58	This study
PVBV-R2–2114R		AGTAGTGTGCTCCCCTAAACAACAAC			
PVBV-R3–1F	RNA3 segment	AGTAGTGATCTCCCAAATCAACATC	1367	56	This study
PVBV-R3–1367R		AGTAGTGTACTCCCAAATCAAACAAC			
PVBV-R4–1F	RNA4 segment	AGTAGTGATCTCCCTTTAACAAC	1494	56	This study
PVBV-R4–1497R		AGTAGTGTACTCCCTTTATCAACTATTC			
PVBV-R5–1F	RNA5 segment	AGTAGTGATCTCCCATCAACAGC	1094	57	This study
PVBV-R5–997R		AGTAGTGTACTCCCATCACAACTAAAC			

⁎ Underlined nucleotides are not of viral origin.

⁎⁎ bp: base pairs.

⁎⁎⁎ Tm: annealing temperature.

@ PVBV-3C: complementary to 3′ terminal end of PVBV genomic RNAs.

### 2.5 Illumina RNA sequencing

The purified total RNA samples were submitted to Novogene Corp., Sacramento, CA, or to the Arizona Genetics Core, Tucson, AZ for high-throughput sequencing (HTS) with the Illumina® HiSeq 2500 or NovaSeq 6000 platform. Following ribosomal RNA depletion, the cDNA libraries were prepared using their standard protocol and sequenced to obtain 150-bp pair-end reads. The quality of raw reads was analyzed using FastQC, available at https://www.bioinformatics.babraham.ac.uk/projects/fastqc/, and reads were trimmed using BBDuk (Department of Energy, Joint Genome Institute, Berkeley, CA). The total number of reads pre- and post-trimming were determined using an in-house script and *de novo* assembled with rnaviralSPAdes software, implemented in SPAdes ver 3.15.4 (Prjibelski et al., 2020). Virus-specific *de novo* assembled contigs were binned (Johansen et al., 2022) and initial emaravirus identification was carried out using BLASTn (Altschul et al., 1990) to query the non-redundant NCBI virus Refseq database, available at https://ftp.ncbi.nlm.nih.gov/refseq/release/viral/. Contigs were assembled on the High-Performance Computing (HPC) cluster in the Research Data Center (RDC) at The University of Arizona.

### 2.6 Amplification and sequencing of full-length palo verde broom virus genome RNA segments

For PVBV isolates that yielded an incomplete sequence for one or more RNA segments, when available, the respective genome segments were RT-PCR amplified, cloned, and sequenced from total RNA used for the corresponding RNAseq analysis. When the RNA had been depleted, total RNA was isolated from leaf samples collected from the same tree, and used as the source for RT-PCR amplification. The first-strand cDNA was synthesized, as described above, followed by incubation with ribonuclease H (RNAse H; New England Biolabs), according to the manufacturer’s instructions. The RT-PCR amplification was carried out using primers (Table 1) designed to anneal to the 5′- and 3′- terminal ends of each RNA genome segment and LongAmp® Hot Start Taq 2X Master Mix (New England Biolabs), according to manufacturer’s instructions. Amplicons of the expected size ∼2.1-kbp (RNA2), ∼1.3-kbp (RNA3), ∼1.5-kbp (RNA4), and ∼1.0 (RNA5) DNA fragment were analyzed on a 1 % agarose gel by electrophoresis in 1X TAE buffer, pH 8.0. The band corresponding to each amplicon was excised from the gel using a sterile razor blade (VWR, Radnor, PA) and gel-purified using Wizard® SV Gel and PCR Clean-Up System (Promega Corp., Madison, WI). The purified amplicons were ligated into the pCR™4-Topo® TA plasmid vector (Invitrogen) and transformed into chemically-competent strain DH5α *E. coli* cells. Plasmid DNA was isolated using Qiagen Plasmid Purification System (Germantown, MD). The plasmids containing cloned inserts were submitted for whole plasmid DNA sequencing using the Oxford Nanopore Technologies nanopore platform (Plasmidsaurus, Louisville, KY; https://plasmidsaurus.com/).

### 2.7 Annotation of palo verde broom virus genomes

Emaravirus coding regions, or predicted open reading frames (ORFs), were identified using Open Reading Frame Finder (ORFfinder) [https://www.ncbi.nlm.nih.gov/orffinder/]. The five genomic segments, ORFs RNA1-RNA5, for the isolates determined here (*n* = 19) sequences available in the GenBank database (*n* = 5; Table 2) were concatenated and analyzed, per below. For isolates found to have two RNA1, RNA2, or RNA5 genome segments (Table 2), all of the predicted ORFs for each isolate were concatenated and aligned to identify the genome segment that shared the greatest homology with and the five apparently cognate RNA genome segments. Sequences of the individual RNA segments and concatenated RNA segments (complete genome) were aligned using Muscle (Edgar, 2004) implemented in Geneious Prime (GraphPad Software LLC, Boston, MA).

**Table 2:** Genome concatenation scheme that was utilized for palo verde broom virus (PVBV) isolates analyzed in this study*.

GenBank accession⁎⁎	Isolate	Host (common name)	Duplicate copy(ies)	RNA1	RNA2	RNA3	RNA4	RNA5
MF766024	P3	*Parkinsonia florida* (blue palo verde)	No	1	2	3	4	5
MF766025	P4	*Parkinsonia florida* (blue palo verde)	No	1	2	3	4	5
MF766026	P5	*Parkinsonia florida* (blue palo verde)	No	1	2	3	4	5
MF766027	P8	*Parkinsonia florida* (blue palo verde)	No	1	2	3	4	5
MF766028	P9	*Parkinsonia florida* (blue palo verde)	No	1	2	3	4	5
PV943489	19.73–11 (BPV1)	*Parkinsonia florida* (blue palo verde)	No	1	2	3	4	5
PV943491	19.134–3 (BPV4)	*Parkinsonia florida* (blue palo verde)	No	1	2	3	4	5
PV943492	21.101–2	*Parkinsonia aculeata* (Mexican palo verde)	No	1	2	3	4	5
PV943493	21.101–4	*Parkinsonia florida* (blue palo verde)	No	1	2	3	4	5
PV943494	21.101–6A	*Parkinsonia florida* (blue palo verde)	Yes (RNA2)	1	**2A**	3	4	5
PV943494	21.101–6B			1	**2B**	3	4	5
PV943495	21.131–1A	*Parkinsonia florida* (blue palo verde)	Yes (RNA1)	**1A**	2	3	4	5
PV943496	21.131–1B			**1B**	2	3	4	5
PV943499	23.27–1A	*Parkinsonia aculeata* (Mexican palo verde)	Yes (genome)	1A	2A	3A	4A	5A
PV943500	23.27–1B			1B	2B	3B	4B	5B
PV943501	23.27–2A	*Parkinsonia florida* (blue palo verde)	Yes (RNA1)	**1A**	2	3	4	5
PV943502	23.27–2B			**1B**	2	3	4	5
PV943503	23.27–4A	*Parkinsonia florida* (blue palo verde)	Yes (genome)	1A	2A	3A	4A	5A
PV943504	23.27–4B			1B	2B	3B	4B	5B
PV943505	23.27–5	*Parkinsonia praecox* (Sonoran palo verde)	No	1	2	3	4	5
PV943507	23.27–11	*Parkinsonia microphylla* (foothills palo verde)	No	1	2	3	4	5
PV943508	23.27–13	*Parkinsonia microphylla* (foothills palo verde)	No	1	2	3	4	5
PV943509	23.27–22	*Parkinsonia florida* (blue palo verde)	No	1	2	3	4	5
PV943510	23.27–42	*Parkinsonia aculeata* (Mexican palo verde)	No	1	2	3	4	5

⁎ For isolates that have duplicate copies of an RNA segment, the individual sequences were concatenated to the remaining ORFs thus creating two scenarios for the full-length genomes (cf. segments in bold font).

⁎⁎ The GenBank accession numbers listed in Table 2 are for RNA1. The accession numbers for RNA2-RNA5 of isolates P3-P9 are, RNA2: MF766029–33; RNA3: MF766034–38; RNA4: MF766039–43; RNA5: OM250026–30. For the accession numbers of RNA2-RNA5 for other isolates, check Table S1B.

### 2.8 Pairwise distance analysis and similarity plots

The sequence demarcation tool (SDT) v1.2 (Muhire et al., 2014) was used to calculate the PVBV nucleotide and amino acid pairwise distances and color-coded heatmaps for the individual and concatenated RNA1–5 genome segments, respectively.

The similarity plots were based on genetic divergence estimates of individual and concatenated RNA1–5 segments using SimPlot (Lole et al., 1999) and Kimura 2-parameter model with sliding window size of 200-bp, a step size of 20-bp, and GapStrip.

### 2.9 Phylogenetic and network analysis

A Bayesian phylogenetic tree was reconstructed for the individual and concatenated RNA genome segments using MrBayes v3.2.7a (Ronquist et al., 2012). The best-fitting evolutionary model was identified as the General Time Reversible (GTR) model with invariable sites and among-sites rate variation with gamma distribution (GTR+*I* + *G*) in PAUP (Wilgenbusch and Swofford, 2003). The Bayesian analysis consisted of two replicates with two chains each for 100 million generations, run on the UA HPC cluster, Research Data Center, The University of Arizona. The tree was drawn with FigTree v1.4.4 (https://github.com/rambaut/figtree/releases), and final editing was carried out using Adobe® Illustrator 2019.

The SplitsTree4 program version 4.14.6 (Huson and Bryant, 2006) was used to evaluate the tree structure for individual and concatenated PVBV RNA1–5 genome segments, in relation to reference emaraviruses, using a non-evolutionary approach that implements the neighbor-net algorithm (Bryant and Moulton, 2004). The evolutionary distances were inferred using Uncorrected_P and the network was drawn using the EqualAngle option. The reticulated network tree option was selected instead of a single bifurcated network tree to reveal contrasting phylogenetic signals and predicted recombination events. The resultant predicted events were substantiated using the pairwise homoplasy index (PHI; Φw) test statistic (Bruen et al., 2006).

### 2.10 Predicted intraspecific recombination

Analysis of PVBV RNA1–5 genome segments for predicted recombination was carried out using seven algorithms, RDP, GENECONV, BootScan, MaxChi, Chimaera, SiScan, and 3Seq methods, implemented in RDP 4.0 (Martin et al., 2015). The default settings were used, except for the general recombination detection options, in which ‘sequences’ were set to linear and the *P*-value threshold was set at *P* < 0.05 with a Bonferroni correction. The predicted recombination events were considered robust e.g. statistically significant (*P* < 0.05), when one or more event, defined by the breakpoints, was predicted by at least five of the seven algorithms.

### 2.11 Genetic structure and selection

The population genetic analysis was carried out for the PVBV RNA1–5 segments using DnaSP v.6.10 (Rozas et al., 2017) to estimate the following parameters: number of haplotypes (h), haplotype diversity (Hd), number of segregating sites (S), total number of mutations (ƞ), average number of nucleotide differences (K), nucleotide diversity (π), synonymous sites (dS), non-synonymous sites (dN), and ratio of non-synonymous sites to synonymous sites (dN/dS).

Haplotype diversity (Hd) of the PVBV RNA1–5 segments for each isolate was analyzed over a range spanning from zero to 1.000 to capture signals ranging from undetectable to high-levels of diversity. In contrast, nucleotide diversity (π) estimates based on a value of zero where variation was undetectable, to 0.100 for extreme divergence (Grant and Bowen, 1998; Nei and Tajima, 1981). Non-synonymous to synonymous nucleotide substitution ratios were estimated using MEGA7 software (Kumar et al., 2016). The test of neutrality was carried out using Tajima’s *D* (Tajima, 1989), and Fu and Li’s D* and F* (Fu and Li, 1993), implemented in DnaSP v.6.10 (Rozas et al., 2017). Selection and per site selection analyses of PVBV coding regions was carried out using Fixed Effects Likelihood (FEL) and Mixed Effects Model of Evolution (MEME) tools, respectively (Murrell et al., 2012; Pond et al., 2005), with a *P*-value of <0.05 considered statistically significant. The selection estimates were inferred using the Fast Unconstrained Bayesian AppRoximation (FUBAR) tool. The average ratio of non-synonymous (dN) and synonymous (dS) substitution rate per site were estimated using the Single-Likelihood Ancestor Counting (SLAC) tool (Pond et al., 2005) for which dN/dS = 1 is considered indicative of neutral or no selection, whereas dN/dS > 1 indicates positive-diversifying selection, and dN/dS < 1 denotes negative-purifying selection. All of the analyses were carried out using Datamonkey 2.0 software (Weaver et al., 2018).

## 3 Results

### 3.1 Palo verde witches broom symptoms, virus detection, and eriophyid mite counts

Among the 70 palo verde trees sampled, 23 trees (32.9 %) were blue palo verde (BPV), 16 (22.9 %) were foothills palo verde (FPV), 14 (20.0 %) were Mexican palo verde (MPV), eight (11.4 %) were Desert Museum palo verde (DMPV), six (8.6 %) were Sonoran palo verde (SPV), and three (4.2 %) were hybrid palo verde (HPV) (Table 3).

**Table 3:** Summary of *Parkinsonia* spp. and hybrid samples with visual witches’ broom symptoms, palo verde broom virus (PVBV) infection status based on RT-PCR assay, and eriophyid mite counts.

Common name	Latin name	Samples No.	Symptomatic plants No. ( %)	PVBV infected plants No. ( %)	Plants with mites (No.)	Eriophyid mites range (No. /plant)
Blue palo verde	*P. florida*	23	19 (82.6.)	21 (91.3)	11/18*	0–16
Mexican palo verde	*P. aculeata*	14	9 (64.2)	14 (100)	8/9	0–50
Foothills palo verde	*P. microphylla*	16	10 (62.5)	10 (62.5)	3/9	0–4
Sonoran palo verde	*P. praecox*	6	0 (0.0)	3 (50.0)	2/6	0–31
Desert Museum palo verde	*P. x* ‘Desert Museum’	8	0 (0.0)	6 (75.00)	7/8	0–40
Hybrid palo verde	*P*. x hybrid	3	0 (0.0)	3 (100)	3/3	100->2000
Total		70	38 (54.3)	57 (81.4)	34/53	

⁎ Number of trees sampled for mites.

Data consisting of the three variables, presence of witches’ broom symptoms, PVBV infection as determined by RT-PCR detection, and presence of eriophyid mites are presented in Table S2A. Data points for all three variables were established for 53 of the 70 (77 %) of the trees sampled, comprising two-thirds blue palo verde trees, and one-third-Mexican palo verde trees.

Symptoms of witches’ broom disease were observed in 38 of 70 (54 %) sampled trees (Table 3). The prevalence of symptoms ranged from 63 % to 83 % in blue palo verde, Mexican palo verde, and foothills palo verde, but no symptoms were observed in Sonoran palo verde or either of the two palo verde hybrids (Table 3). PVBV infection was assessed by RT-PCR amplification of the PVBV NP gene (Table 1). Based on the results of RT-PCR amplification, 57 of 70 trees (81 %) were positive for PVBV infection, indicating that all four palo verde species and the two palo verde hybrids are PVBV hosts. The infection rates were lowest in Sonoran palo verde (50 %) and foothills palo verde (62 %), respectively, while the remaining taxa showed PVBV infection rates of 75 % to 100 % (Table 3). Blue palo verde had the highest PVBV infection prevalence (detection), at 91 %, with the greatest number of symptomatic trees, at 83 % (Table 3).

The four palo verde species and the two hybrids differed with respect to the three variables: witches’ broom symptoms, PVBV infection, and palo verde mite presence (Table S2A-D). Of the 53 trees for which data for all three variables were documented, 11 trees were positive for all three variables (Table S2A). This comprised two-thirds of the blue palo verde trees and one-third of the Mexican palo verde trees. The largest number of trees (16) exhibited no witches’ broom symptoms, but harbored detectable PVBV infection and were colonized by mites (Table S2A). These variables were common to the four palo verde species and the two hybrids. Trees that were PVBV-positive, harbored no mites, and either did or did not exhibit witches’ broom symptoms included the blue, foothills, and Sonoran paloverde species (Tabe S2A). In contrast, neither of the hybrids developed broom symptoms even though most were positive for PVBV infection and harbored eriophyid mites.

About half of the trees exhibited either witches’ broom symptoms or harbored eriophyid mites and were positive for PVBV detection (Table S2A, S2B). Approximately one-third of these trees were PVBV-positive but harbored no mites and had no witches’ broom symptoms. Among the three symptomatic palo verde species, about half (11/20; 55 %) were colonized by eriophyid mites, while eriophyid mite infestations were recorded on 72 % (21/29) of trees that exhibited no witches’ broom symptoms (Table S2C).

In summary, the palo verde species and/or hybrids studied here showed variable responses to symptom development, PVBV infection, and eriophyid mite infestation (Table S2A-D). Some palo verde species were either PVBV-positive or -negative, and harbored no eriophyid mites, while also being asymptomatic (Table S2A-D). The two hybrids showed no broom symptoms, but harbored eriophyid mites, or were positive for PVBV infection or both.

### 3.2 Eriophyid mite identification and mite counts on palo verde trees

**Fig. 1:**
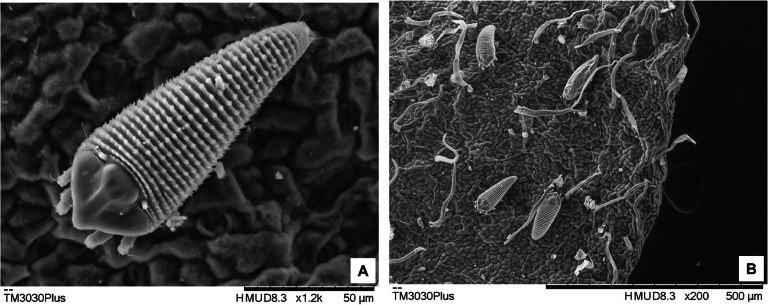
Electron micrographs of the palo verde eriophyid mite - *Aculus cercidii* Keifer 1965. Dorsal aspect of an adult palo verde eriophyid mite at 1.2 K magnification. The bar is 50 µm in length (A.), population of palo verde eriophyid mites on the surface of a leaflet including; a nymph, a molting immature, an adult, an egg and an exuvia at 200x magnification. The bar is 500 µm in length (B.). (Courtesy, Dr. R. Ochoa and A. Ulsmer, USDA-ARS Systematic Entomology Laboratory).

Subsets of leaf samples collected from symptomatic blue palo verde trees were examined using a dissecting light microscope for presence or absence of the palo verde eriophyid mite (Table 3). The key morphological characters were examined by SEM and compared with those reported by Keifer et al. (1982) and available notes (R. Ochoa and A. Ulsamer, USDA Systematic Entomology Laboratory, Beltsville, MD) (Fig. 1A), which resulted in identification of the mites, as the palo verde mite (Fig. 1B). To corroborate the identification specific characters reported in the literature (Baker, 1996; Keifer, 1982) (Fig. 1B) mites from the same subsets were mounted on carbon sticky tabs and examined under a Hitachi TM3030 scanning electron microscope (SEM).

Eriophyid mites were found on 64 % of trees for which mite counts were taken (Table 3). When present, mites were most often found colonizing the abaxial side and the bases of leaflets and petioles. The eriophyid mite counts among the palo verde species ranged from four (4) mites on foothills palo verde, to more than 2000 mites on hybrid palo verde (Table 3). On the Desert Museum hybrid palo verde, the mite counts ranged from 40 per tree to greater than 2000 mites per tree on the other palo verde hybrid, which showed no visible broom symptoms but tested positive for PVBV infection. The number of mites counted on leaf samples from the other four palo verde species ranged from zero to 50 (Table 3).

### 3.3 High throughput sequence reads and assembly of genome segments

The Illumina® raw reads were *de novo* assembled using the pipeline, as described above. The closest matches were identified using BLASTn to search the virus RefSeq database, downloaded from the NCBI reference sequence database. The raw read counts ranged from 115,113,468 to 1108,382,834 (Table S1A). For *de novo* assembled contigs, the number of reads remaining after the quality-filtering step ranged from 115,046,462 to 1107,766,256 reads. The k-mer coverage of reads mapping to PVBV RNA1–5 genome segments ranged from 1.674 (RNA2) to 122,098.2 (RNA3) (Table S1A).

Sixteen full-length PVBV genomes were assembled from twenty-nine RT-PCR positive virus-infected samples representing all four *Parkinsonia* spp. and two hybrid trees. For the three isolates that yielded only a partial PVBV genome, and the suspect ‘missing’ genome segments, typically, RNA2–5, were amplified from total RNA by high fidelity PCR amplification. The RNA genome sequences were deposited in the GenBank database and assigned the accession numbers PV943489-PV943616 (Table S1B). Among them were isolates similar and representative of previously published sequences, 23.27–1 (MPV, TUC) and 23.27–4 (BPV, TUC), whereas, several previously unpublished variants were identified, 23.27–1A and 23.27–1B, and 23.27–4A and 23.27–4B (Table 2). Finally, two copies of RNA2 and RNA5 were recovered for isolates 21.101–6 (BPV, TUC) and 21.101–8 (MPV, TUC), respectively, all subjected to confirmatory RT-PCR amplification and confirmatory sequencing.

The size of the RNA genome segments recovered were: for RNA1: 6985–7068 nt, RNA2: 2094–2111 nt, RNA3: 1186–1368 nt, RNA4: 1429–1507 nt, and RNA5: 907–1077 nt. The length and sequence for both terminal ends of the RNA2-RNA5 segments were confirmed by RT-PCR amplification, whole plasmid sequencing, followed by multiple sequence alignment with all available PVBV reference sequences (NC_078374–77; OM250028). The PVBV genome segments and lengths, respectively, were for RNA2: 2113–2114 nt; for RNA3: 1367–1370 nt, for RNA4: 1490–1507 nt, and for RNA5: 1070–1079 nt, respectively. The lengths of assembled, concatenated RNA1–5 ORFs ranged from 11,439–11,445 nt.

### 3.4 Pairwise nucleotide and amino acid sequence comparisons

#### 3.4.1 Pairwise nucleotide sequence identity analyses

**Fig. 2:**
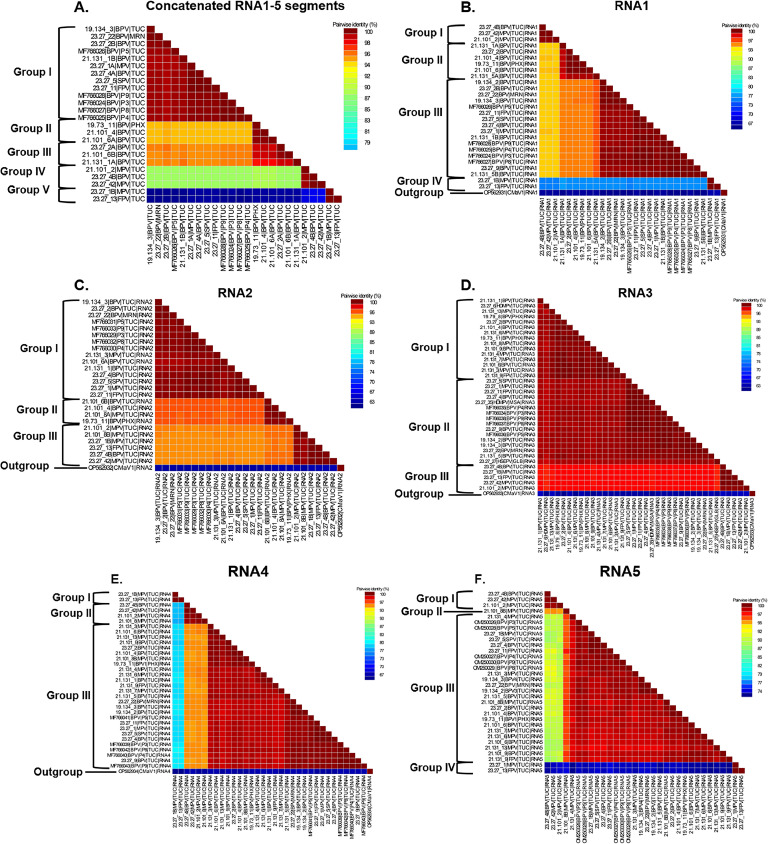
Pairwise nucleotide (nt) sequence distance analysis of Palo verde broom virus (PVBV) concatenated genome sequences (A.), RNA1 genome segment (B.), RNA2 genome segment (C.), RNA3 genome segment (D.), RNA4 genome segment (E.), and RNA5 genome segment (F.). The distance matrix comprise isolates determined in this study and PVBV sequences in GenBank. Multiple sequence alignment was carried out in MUSCLE (Edgar, 2004), implemented in the Sequence Demarcation Tool (SDT) (Muhire et al. 2014). The *Parkinsonia* spp. hosts were abbreviated as: Blue palo verde (BPV), Foothills palo verde (FPV), Mexican palo verde (MPV), and Sonoran palo verde (SPV). The geographic locales were abbreviated as: Tucson (TUC), Marana (MRN), and Phoenix (PHX).

The concatenated PVBV ORFs (*n* = 24) shared 78.3–100 % pairwise nucleotide sequence identity with one another, and for five distinct groups based on phylogenetic and pairwise distances analyses (Fig. 2A-F). Group I contained PVBV isolates (*n* = 13) from: BPV (19.134–3, 21.131–1B, 23.27–2, 23.27–22, 23.27–4A, P3, P4, P5, P8, P9), FPV (23.27–11), MPV (23.27–1A), and SPV (23.27–5), which shared 78.4–94.4 % nt identity with the other four lineages. Group II isolates (*n* = 3) (19.73–11, 21.101–4, 21.101–6A) were exclusively associated with BPV trees, and shared relatively high nt identity with the four other groups, at 93–93.2 %. All group III isolates (*n* = 3) were recovered from BPV (23.27–2A, 21.101–6B, 21.131–1A), and shared relatively high nt identities, at 94.2–94.5 % with the other four groups of PVBV isolates. Group IV contained three isolates, one from BVP (23.27–4B) and two from MPV (21.101–2, 23.27–42) trees, which shared 89.6–89.8 % nt identity with all other isolates. Group V contained isolates, 23.27–1B (MPV) and 23.27–13 (FPV), which shared relatively low nt identity with all other isolates, at 78.4–80.3 %, respectively.

Analysis of individual RNA1–5 genome segments at the nucleotide level indicated that the RNA1 shared 73–100 % identity, while RNA2–5 shared 89–100 %, 93–100 %, 75–100 %, and 73–100 % nt identity, respectively. Also, the full-length genomes of PVBV isolates 23.27–1 (MPV, TUC) and 23.27–4 (BPV, TUC), were highly variable, with shared nucleotide identities of 73.5–100 % nt, respectively (Table 4). Two full-length RNA1 genome segments were detected in PVBV-infected BPV, 21.131–1 (BPV, TUC), 21.131–5 (BPV, TUC), and 23.27–2 (BPV, TUC), and the isolates shared 90–93 % nt identity with each other. In addition, two divergent PVBV genomes were recovered, one each from Mexican palo verde and foothills palo verde, isolates 23.27–1B (RNA5) and 23.27–13 (RNA3), respectively, that exhibited the lowest nt identities across the analogous PVBV RNA5 and RNA3 segments of all PVBV isolates, respectively, of 75 % and 94 % identity, a divergence of 25 % and 6 %. In another scenario, two divergent RNA2 segments were recovered from PVBV isolate 21.101–6 (BPV, TUC) that were 91 % identical, while two divergent RNA5 segments associated with PVBV isolate 21.101–8 (MPV, TUC) shared 95 % identity, respectively.

**Table 4:** Genome comparisons of isolates 23.27–1 (MPV) and 23.27–4 (BPV) comprising two genome segments.

	RNA segment(s) nucleotide identity ( %)				
Nucleotide comparison(s)*	RNA1	RNA2	RNA3	RNA4	RNA5
23.27–1A vs. 23.27–1B	73.9	89.6	94.8	76.9	74.4
23.27–1A vs. 23.27–4A	99.7	100.0	100.0	100.0	99.9
23.27–1A vs. 23.27–4B	88.8	89.7	94.8	90.2	86.8
23.27–1B vs. 23.27–4A	73.8	89.6	94.8	76.9	74.3
23.27–1B vs. 23.27–4B	73.5	100.0	99.9	75.9	73.8
23.27–4A vs. 23.27–4B	88.5	89.7	94.8	90.2	87.4

	RNA segment(s) amino acid identity ( %)⁎⁎				
Amino acid comparison(s)	RdRp	GP	NP	MP	HP
23.27–1A vs. 23.27–1B	77.8	94.8	99.7	87.7	70.2
23.27–1A vs. 23.27–4A	99.7	100.0	100.0	100.0	100.0
23.27–1A vs. 23.27–4B	97.5	95.0	99.3	98.1	96.1
23.27–1B vs. 23.27–4A	77.7	94.8	99.7	87.7	70.2
23.27–1B vs. 23.27–4B	77.6	99.8	99.7	87.7	69.7
23.27–4A vs. 23.27–4B	97.3	95.0	99.3	98.1	96.1

⁎ Isolates designated as genome A have a greater nucleotide identity to palo verde broom virus (PVBV) reference sequence (NC_078374–7; OM250027) compared to genome B that has a lower nucleotide identity.

⁎⁎ RdRp: RNA-dependent, RNA polymerase; GP: glycoprotein; NC: nucleocapsid protein; MP: movement protein; and HP: hypothetical protein.

#### 3.4.2 Pairwise amino acid sequence identity analyses

The amino acid (aa) similarities of predicted PVBV ORFs spanned 68–100 %. The aa similarities for RdRp comparisons among all PVBV isolates was 78–100 %, followed by the GP, NP, MP, and HP, at 93–100, 97–100, 86–100, and 68–100 aa percent identity. Consistent with the nt sequence identity comparisons, PVBV isolates 23.27–1B and 23.27–13 shared the lowest aa identity, with one exception that involved NP, for which isolates 23.27–6 from DMPV and 23.27–27 from HSEPV shared the least aa similarity with all other isolates. For PVBV isolates identified as harboring two full-length genomes, the predicted ORFs shared very low aa identities, of 69.7–100 % between each other (Table 4). For isolates 21.131–1 (BPV, TUC), 21.131–5 (BPV, TUC), and 23.27–2 (BPV, TUC), two RdRp ORFs were identified that shared 98.4–98.6 % identity. Finally, two GP ORFs were recovered for isolate 21.101–6 (BPV, TUC) that were 94 % identical, and two HP ORFs were recovered from PVBV isolate 21.101–8 (MPV, TUC) that shared 98.7 % aa identity.

#### 3.4.3 Sliding window analysis

**Fig. 3:**
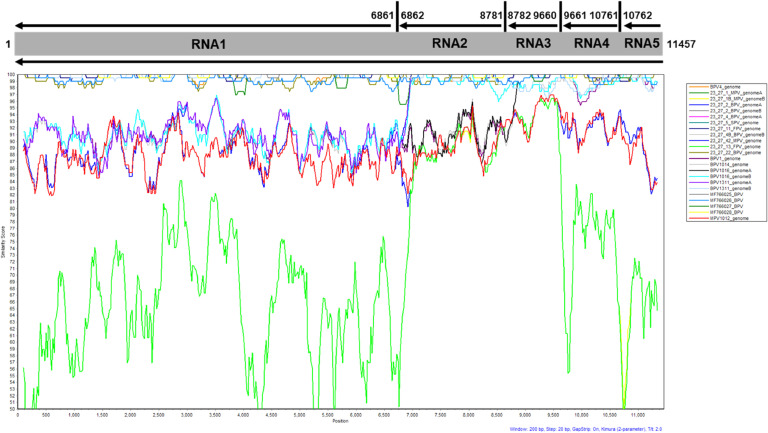
Nucleotide sequence divergence and similarity plot of the concatenated open reading frames of palo verde broom virus (PVBV) isolates. The similarity plot was carried out using SimPlot (Lole et al. 1999) with Kimura 2-parameter model using a window size of 200 base pairs (bp) and a step size of 20 bp. The concatenated sequence of reference isolate, P4 (NC_078374–77; OM250028) was selected as the query sequence.

The SimPlot sliding window analysis showing the nucleotide divergence among the PVBV RNA segments showed that isolate 23.27–13 (FPV, TUC) was the most divergent among all other PVBV isolates, with respect to the RdRp, movement protein (MP) and hypothetical protein (HP) (Fig. 3), with similarity scores ranging from 50–84 %. The divergence among the other PVBV isolates ranging from highest to lowest, was 21.101–2 (MPV, TUC), 21.101–6B (BPV, TUC), 21.131–1A (BPV, TUC), 21.101–4 (BPV, TUC), and 21.101–6A (BPV, TUC), the latter, of which encodes the most divergent glycoprotein gene among the PVBV isolates studied here.

### 3.5 Bayesian phylogeny

**Fig. 4:**
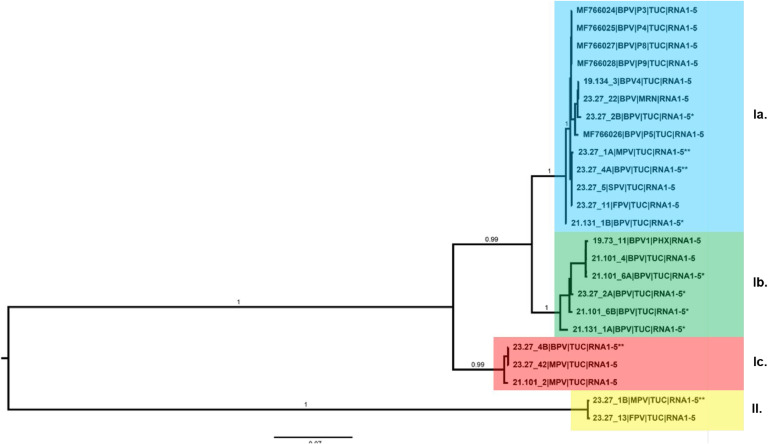
Bayesian phylogenetic analysis of palo verde broom virus (PVBV) RNA1 to RNA5 genome segment open reading frames (ORFs). Multiple sequence alignment was carried out using MUSCLE (Edgar, 2004), implemented in Geneious Prime (GraphPad Software, LLC, Boston, MA). The evolutionary history was inferred using MrBayes 3.2.7a (Ronquist et al. 2012) with 100,000,000 generations (sampling freq=5000; nruns=2, nchains=2) and a burn-in of 2500 generations. Evolutionary distances were estimated using the General Time Reversible (GTR) model, and invariable sites (I), with the rate variation among sites modeled with a gamma distribution model (GTR+*I* + *G*). Evolutionary analysis was carried out on the University of Arizona High Performance Computing (HPC) cluster, UA Research Data Center. The consensus tree was drawn with Fig Tree v1.4.4 (http://tree.bio.ed.ac.uk/software/figtree/) and edited in Adobe Illustrator 2024. The *Parkinsonia* spp. hosts were abbreviated as: Blue palo verde (BPV), Foothills palo verde (FPV), Mexican palo verde (MPV), and Sonoran palo verde (SPV). The geographic locations were abbreviated as: Tucson (TUC), Marana (MRN), and Phoenix (PHX).

**Fig. 5:**
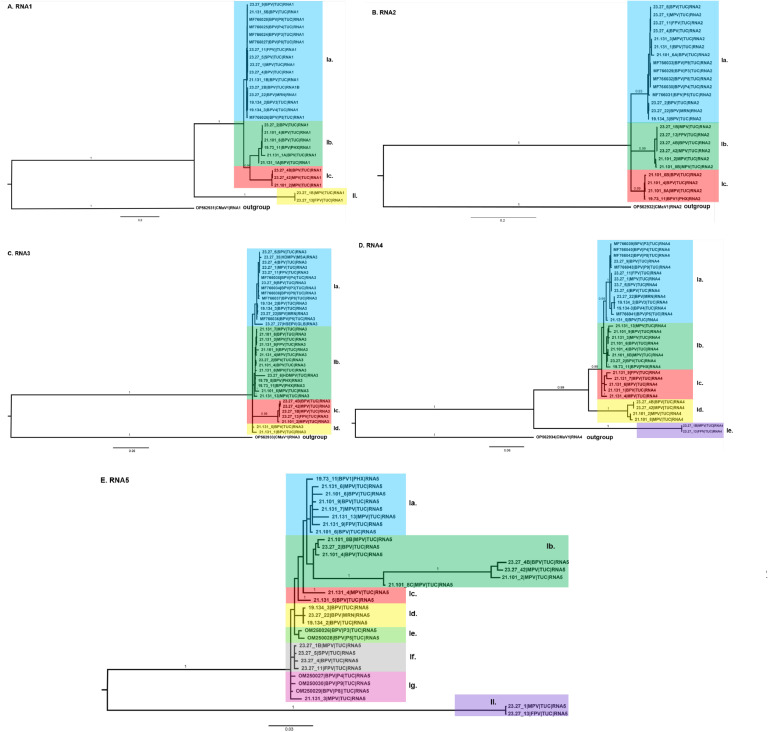
Bayesian phylogenetic analysis of palo verde broom virus (PVBV) RNA1 (A), RNA2 (B), RNA3 (C), RNA4 (D), and RNA5 (E) genome segments. Multiple sequence alignment was carried out using MUSCLE (Edgar, 2004), implemented in Geneious Prime (GraphPad Software, LLC, Boston, MA). The evolutionary history was inferred using MrBayes 3.2.7a (Ronquist et al. 2012) with 50,000,000 generations (sampling freq=5000; nruns=2, nchains=2) and a burn-in of 2500 generations. Evolutionary distances were estimated using the General Time Reversible (GTR) model and invariable sites (I), with the rate variation among sites estimated using a gamma distribution model (GTR+*I* + *G*). Evolutionary analysis was carried out on the University of Arizona High Performance Computing (HPC) cluster, UA Research Data Center. The consensus tree was drawn with Fig Tree v1.4.4 (http://tree.bio.ed.ac.uk/software/figtree/) and edited in Adobe Illustrator 2024. Outgroup: Callicarpa mosaic-associated virus 1 (CMaV1). The *Parkinsonia* spp. host species are abbreviated as: Blue palo verde (BPV), Foothills palo verde (FPV), Mexican palo verde (MPV), and Sonoran palo verde (SPV). Geographic location abbreviations are: Tucson (TUC), Marana (MRN), and Phoenix (PHX).

The Bayesian phylogeny for the concatenated and individual genome segments of PVBV isolates reported from this study, and reference GenBank isolates, respectively, are shown in Fig. 4, Fig. 5A-E. The callicarpa mosaic-associated virus 1 (CMaV1) RNA1–4 (OP562931–34) was used as the outgroup.

#### 3.5.1 Concatenated PVBV genome segments

The concatenated segments RNA1–5 formed two distinct clades, I and II, each having robust support, e.g. posterior probability of 1.0 (Fig. 4). Clade I consisted of two sub-clades, with robust support of 0.99 posterior probability. Sub-group Ia was represented by five previously characterized PVBV genome sequences (Avelar et al., 2018; Ilyas et al., 2018) determined for isolates collected from symptomatic BPV trees in 2015 when PVBV was first discovered. Subclade Ib, contained PVBV isolates from BPV, FPV, MPV, and SPV recovered in 2019, 2021, and 2023, and the third subclade, Ic, contained three PVBV isolates recovered from BPV and MPV in 2021 and 2023. For the clade II isolates, the two most divergent members of clade II were isolated from BPV and MPV species, respectively.

#### 3.5.2 Individual PVBV RNA genome segments

The RNA1 genome segments formed two clades, I and II (Fig. 5A). Clade I consisted of three sub-groups of isolates representing four palo verde species, BPV, FPV, MPV, and SPV, compared to clade II, whose isolates represented only palo verde species, BPV and MPV. All of the RNA2 genome segments grouped in the same clade and formed three sub-groups with a posterior probability support of 0.93, 0.99, and 0.99, respectively (Fig. 5B). Similarly, the RNA3 segments clustered in one clade that clustered as three subgroups and two divergent outliers, with a robust posterior probability support, at 1.0, respectively (Fig. 5C). The RNA4 segments formed two distinct clades, I and II, with robust support of 1.0 posterior probability (Fig. 5D). Clade II was divided into subgroups I and II. Sub-group I contained isolates from four palo verde species, BPV, FPV, MPV, and SPV collections from 2015, 2019, 2021, and 2023, respectively, while sub-group II contained RNA4 sequences from only BPV and MPV tree collections from 2021 to 2023, respectively. The PVBV RNA4 isolates grouping in clade II originated from the 2023 FPV and MPV sample collections. Finally, the RNA5 segment formed two clades with robust support, at 1.0 posterior probability, respectively (Fig. 5E). The RNA5 clade I isolates were highly divergent, forming seven sub-groups of PVBV isolates sequenced from BPV, FPV, MPV, and SPV trees during 2015, 2019, 2021, and 2023. In contrast, clade II contained two isolates, both collected in 2023, and represented one each from FPV and MPV.

### 3.6 Population structure based on network analysis

**Fig. 6:**
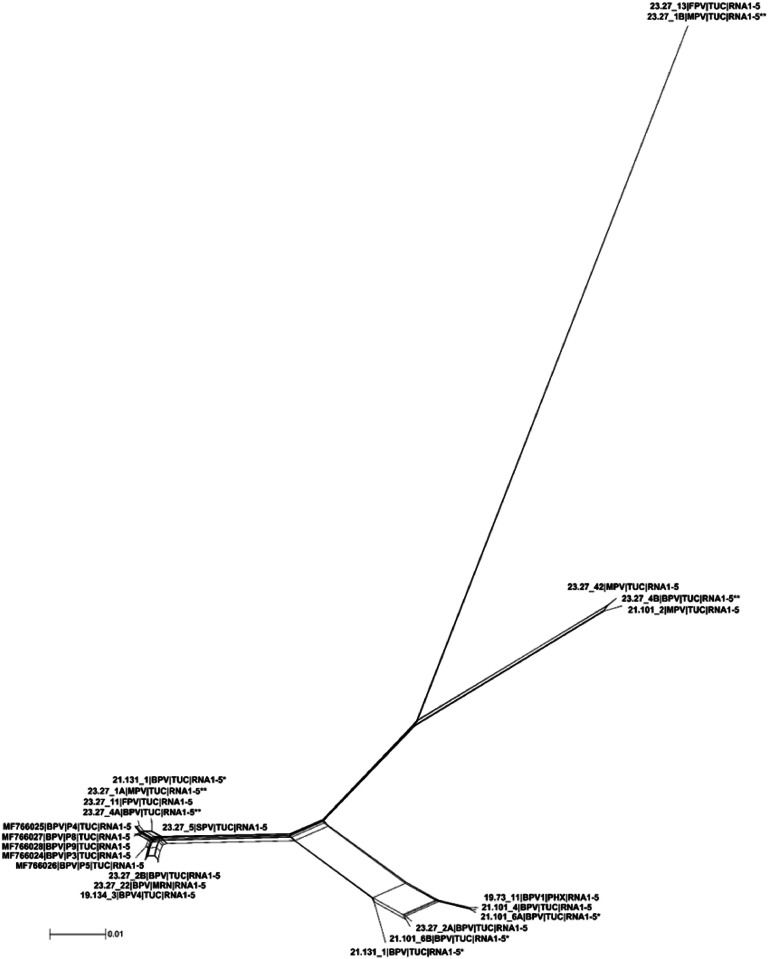
Phylogenetic network analysis of palo verde broom virus (PVBV) genome sequences of isolates studied here and reference sequences available in the GenBank database. The analysis was carried out using the NeighborNet algorithm implemented in SplitsTree4 v4.14.6 (Huson and Bryant, 2006). The tree was reconstructed using Uncorrected_P method, and the network was drawn using EqualAngle. The bifurcating paths indicate a low probability of recombination. The host species abbreviations are: blue palo verde (BPV), foothills palo verde (FPV), Mexican palo verde (MPV), Sonoran palo verde (SPV) geographic locations are abbreviated as follows: Alabama (AL), Argentina (AR), Brazil (BR), China (CN), Florida (FL), Georgia (GA), Louisiana (LA), North Carolina (NC), Oklahoma (OK), South Carolina (SC), South Korea (KR), and Texas (TX).

Network analysis of individual PVBV genome segments RNAs1–5, as well as the concatenated ORFs, produced a reticulated tree consisting of four clusters (Fig. 6 and Figure S1A-E), and probable occurrences of prior recombination (Figure S1A-E). The predictions for the concatenated ORFs RNA1 (*P* = 0.000), RNA3 (*P* = 0.014), and RNA5 (*P* = 5.218E-15) were confirmed as robust, based on PHI, were statistically significant. Although recombination was predicted for RNA2 (*P* = 0.1215) and RNA4 (*P* = 0.816), these events were not statistically significant.

### 3.7 Predicted recombination

**Fig. 7:**
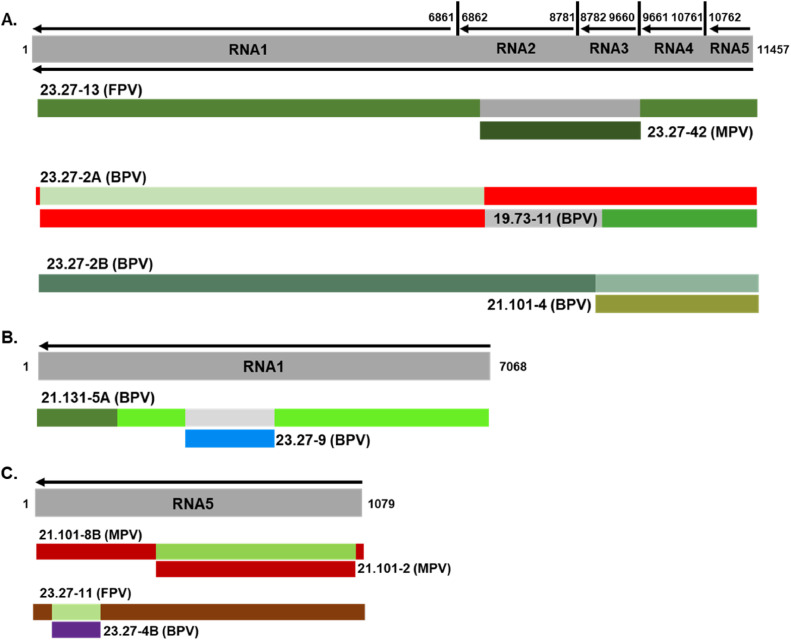
Schematic illustration of predicted recombination events in the palo verde broom virus (PVBV) genome (A.) concatenated genome segments, (B.) RNA1 genome segment, and (C.) RNA5 genome segment. The analysis was carried out in Recombination Detection Program (RDP) v4.83.0, (Martin et al., 2015) using defaults parameters except that the sequences were set to linear under the general recombination detection options.

Recombination analysis using RDP4 identified predicted recombination events in the RNA1–5, and the concatenated ORFs1–5 of PVBV isolates. Recombination was predicted in the RNA1 of PVBV isolate 21.131–5A sequenced from a BPV tree host, with breakpoints spanning nucleotides 2188 to 3638 (Table 5; Fig. 7). The major parent was identified as isolate 21.101–6 from BPV, while isolate 23.27–9, also from BPV, was identified as the minor parent. The average *P*-values ranged from 4.041 × 10^−14^ to 3.756 × 10^−67^ for the seven algorithms, indicating the identified recombination events were significant. Two recombination events in RNA5 were predicted (Fig. 7) for isolate 21.101–8B from MPV, based on breakpoints located at nucleotides 331 and 1061 (Table 5). The *P*-values for the seven algorithms ranged from 8.495 × 10^−09^ to 1.483 × 10^−30^. The predicted major and minor parents were identified as PVBV isolate 21.101–2 and isolate 21.101–8A, both from MPV. A second RNA5 recombinant was identified for PVBV isolate 23.27–11 from FPV, albeit one breakpoint was undetermined, but the other was identified as nucleotide 225 (Table 5). The major and minor parents were traced to PVBV isolate 21.131–5 and isolate 23.27–4B, respectively, both from BPV. The average *P*-values for the seven algorithms ranged from 2.627 × 10^−03^ to 1.585 × 10^−10^.

**Table 5:** List of palo verde broom virus isolates identified as recombinants sequences.

Sequence	Event	Breakpoint position(s)			Parental isolate(s)		Recombination Detection Method*						
		Begin	End	Recombinant isolate⁎⁎	Minor parent	Major parent	R	G	B	M	C	S	3S
Concatenated	1	6862	9660	23.27–13 (FPV)	23.27–42 (MPV)	Unknown; 21.101–6B⁎⁎⁎ (BPV)	3.1 × 10^−157^	1.38 × 10^−161^	2.29 × 10^−43^	9.29 × 10^−35^	3.29 × 10^−34^	1.82 × 10^−116^	3.10 × 10^−13^
	2	22	6859	23.27–2A (BPV)	19.73–11 (BPV)	23.27–2B (BPV)	1.32 × 10^−50^	9.54 × 10^−31^	1.67 × 10^−47^	4.37 × 10^−26^	4.50 × 10^−08^	2.29 × 10^−68^	4.05 × 10^−128^
	3	8872	11,436	23.27–2B (BPV)	21.101–4 (BPV)	23.27–22 (BPV)	7.28 × 10^−14^	1.10 × 10^−40^	1.59 × 10^−10^	2.56 × 10^−11^	3.92 × 10^−11^	1.36 × 10^−12^	4.35 × 10^−31^
RNA1	1	2188	3638	21.131–5A (BPV)	23.27–9 (BPV)	21.101–6 (BPV)	2.65 × 10^−68^	3.76 × 10^−67^	NS	3.66 × 10^−22^	1.49 × 10^−22^	1.22 × 10^−24^	4.04 × 10^−14^
RNA5	1	331	1061	21.101–8B (MPV)	21.101–8A (MPV)	21.101–2 (MPV)	6.07 × 10^−11^	9.27 × 10^−11^	8.49 × 10^−09^	1.17 × 10^−13^	2.52 × 10^−10^	1.92 × 10^−21^	1.48 × 10^−30^
	2	Undetermined	225	23.27–11 (FPV)	Unknown; 23.27–4B (BPV)	21.131–5 (BPV)	1.59 × 10^−10^	2.24 × 10^−09^	6.76 × 10^−08^	1.92 × 10^−02^	2.63 × 10^−03^	NS	2.71 × 10^−07^

⁎ Recombination detection methods implemented in RDP4: R-RDP; G-GENECONV; B-BOOTSCAN; M-MAXCHI; C—CHIMERA; S-SISCAN; 3S-3SEQ. The statistical support for each method is included.

⁎⁎ BPV-blue palo verde; FPV-foothills palo verde; MPV-Mexican palo verde.

⁎⁎⁎ Isolates with a letter indicate isolates that have more than one copy of a full-length genome or genome segments.

Based on analysis of the concatenated genome sequences, recombination was predicted in RNA1 (*n* = 1) of isolate 23.27–13 (FPV), and in RNA5 (*n* = 2) of PVBV isolates 23.27–2A and 23.27–2B (BPV), respectively. These predictions were supported by all seven algorithms, indicating they were statistically significant (Table 5; Fig. 7). For the recombinant identified as PVBV isolate 23.27–13, the breakpoints were located at nucleotides 6862 and 9660 (Table 5), and the average *P*-value for the seven algorithms ranged from 6.921 × 10^−157^ to 3.108 × 10^−13^. Although the predicted major parent could not be identified with great certainty, isolate 21.101–6B from BPV was identified as a possible major parent, while isolate 23.27–42 from MPV, was identified as the minor parent. For the second recombinant, PVBV isolate 23.27–2A, the breakpoints were located at nucleotide 22 and 6859 (Table 5), respectively, with the minor parent stemming from BPV isolate 19.73–11, and the major parent traced to an isolate 23.27–2B from BPV. The average *P*-value range for the seven algorithms was 6.125 × 10^−82^ to 4.503 × 10^−08^. The third recombinant was identified as isolate 23.27–2B from BPV. The beginning breakpoint was located at nucleotide 8872 to an ending breakpoint at nucleotide 11,436 (Table 5), with the major parent being isolate 23.27–22 from BPV, and isolate 21.101–4 from BPV, as the minor parent. The average *P*-value range for the seven algorithms was 2.044 × 10^−15^ to 5.345 × 10^−07^.

### 3.8 Genetic diversity

Genetic variation and selection were analyzed separately for each PVBV genome segment. The haplotype diversity (Hd) estimated for all five PVBV genomic RNA segments ranged from 0.960 for RNA2 and RNA3, to 0.980 for RdRp (RNA1) (Table 6). The nucleotide diversity (π) ranged from 0.0221 (RNA3, NP) to 0.0846 (RNA1, RdRp), whereas, number of segregating sites (S) ranged from 122 in NP to 2304 for RdRp (Table 6). The total number of mutations (ƞ) ranged from 126 for NP to 2586 for RdRp, while the average number of nucleotide differences was lowest for NP, at 25, and the greatest for RdRp, at 578.2. The non-synonymous and synonymous (dN/dS) ratios for the RNA1–5 codons were <1 for five genome segments, indicating they are evolving under negative selection (Table 6).

**Table 6:** Estimates of population genetic parameters and neutrality tests calculated for the encoded genes of palo verde broom virus (PVBV) palo verde.

Gene	n	h	Hd	S	ƞ	K	π	dS	dN	dN/dS (±*S*.E.)	Tajima’s D	Fu & Li’s D	Fu & Li’s F
RdRp	27	21	0.980	2304	2586	578.2	0.0846	0.0032	0.1062	0.0730±0.0035	−0.55035 ns	1.75149**	1.17288 ns
GP	25	17	0.960	316	322	110.7	0.0577	0.0285	0.0716	0.0431±0.0066	1.19411 ns	1.13057 ns	1.35545 ns
NC	36	21	0.960	122	126	25.36	0.0221	0.0031	0.0288	0.0256±0.0041	−0.53862 ns	−0.91000 ns	−0.92785 ns
MP	33	24	0.973	336	363	56.87	0.0517	0.0143	0.0775	0.0632±0.0051	−1.39755 ns	1.10198 ns	0.28403 ns
HP	32	22	0.970	256	280	40.43	0.0589	0.0290	0.0704	0.0432±0.0072	−1.60974 ns	0.71826 ns	−0.09272 ns

^@^*n* = sample size, *h* = no of haplotypes, Hd = haplotype diversity, *S* = no of segregating sites; ƞ (Eta) = total no of mutations, *k* = average no of nucleotide differences between sequences, Pi (π) = nucleotide diversity, dS = synonymous sites, dN = non-synonymous sites, dN/dS = ratio of non-synonymous nucleotide diversity to synonymous nucleotide diversity.

ns - not significant, *P value < 0.05; **P value < 0.01; ***P value < 0.001.

Potential selection acting at the complete PVBV genome level or on the individual RNA1–5 segments was analyzed separately. The Tajima’s *D*, and Fu and Li’s D* and F* test statistics that are used to predict departures from neutral patterns of selection, were variable and highly dependent on the specific RNA segment. The Tajima’s *D* test results showed negative selection with no statistical significance except for the RNA2 segment, which encodes the virus glycoprotein (Table 6). The Fu and Li’s D* and F* test statistics were non-significant for all segments except for RNA1, which encodes the RdRp, indicating statistically significant, positive selection. The negative Tajima’s *D*, and Fu and Li’s D* and F* test statistics indicated a violation of neutrality associated with positive selection. This pattern is considered reminiscent of rare variants present at low- frequencies possibly attributed to recent founder events, population expansion, or positive selection. In contrast, segments with a positive test statistic are considered reflective of an excess of intermediate-frequency or ancestral variants that were selected in the past through balancing selection or population bottlenecks.

Selection acting on coding regions inferred by four methods, FEL, MEME, FUBAR, and SLAC methods (Table S3), varied depending on the PVBV RNA segment. The FEL program identified codons (aa) undergoing negative selection in all five ORFs. The number of nucleotides ranged from a low 14 in the NP gene to 969 in RdRp. The MEME program identified four, three, and one codon undergoing positive selection in the GP, RdRp, and MP genes, respectively (Table S3). The FUBAR analysis identified both positive and negative selection acting on the codons, including two and one each in the RdRp and NP gene, respectively. The latter analysis also identified sites undergoing negative selection involving SNPS 23 and 1891 in the NP and RdRp, respectively (Table S3). All three SLAC analyses identified three nt undergoing negative selection in NP and HP, compared to 340 nt in RdRp, respectively (Table S4).

## 4 Discussion

Different factors are known to influence the emergence, rate of diversification, and spread of RNA viruses, which can involve mutation rate, extent of recombination, type and population size of the arthropod vector, as well as environmental changes, including those associated with agronomic practices (Zhao et al., 2019). Viruses with an RNA genome tend to have a large population size, short generation times, a propensity for high genetic variation, and evolve rapidly, accumulating mutations faster than most DNA viruses (Duffy, 2018).

Palo verde trees belong to the *Fabacea*e and are native to and widely distributed in the Sonoran Desert. Palo verde trees and shrubs contribute diverse functions in to the flora and fauna of the desert environment (Schuch and Kelly, 2012) and are extremely important for soil conservation by preventing water and wind erosion (Bowers and Turner, 2001; Schuch and Kelly, 2012). Although it is not known when the palo verde trees in the Sonoran Desert encountered PVBV or were first colonized by the palo verde eriophyid mite, the virus-vector complex appears to have been associated with the trees for some time. Their joint interaction is thought to be required for the development of witches’ broom symptoms, which also appears to stimulate feeding and increased reproduction of the palo verde eriophyid mite, collectively, scenarios that point to a deep co-evolutionary history.

The increased prevalence of witches’ broom disease in urban palo verde trees in southern Arizona has spurred the development of the first molecular PVBV assay to facilitate broom disease diagnosis in palo verde trees. Here, seventy palo verde trees representing four *Parkinsonia* species and two hybrids were sampled from 2019 to 2023 and tested for the presence or absence of PVBV RNA3 by RT-PCR detection. The availability of this sensitive, optimized molecular detection test has facilitated the identification of multiple palo verde species as PVBV hosts for the first time, following the initial sequencing of PVBV from broom-symptomatic blue palo verde trees (Ilyas et al., 2018).

Among the 70 trees sampled 38 (54.3 %) showed characteristic witches’ broom disease symptoms. Infection by PVBV was confirmed for 57 trees (81.4 %) by RT-PCR amplification, cloning, and confirmatory sequencing. The results showed that PVBV was detectable in trees representing four palo verde species and two hybrids tested. The blue palo verde trees had the highest rate of infection, at 18 of 23 (91.3 %) while the Sonoran palo verde trees had the lowest infection rate, at three of six (50.0 %). The moderate to high infection frequency observed indicates a strong association between PVBV infection and witches’ broom disease symptoms and was consistent with PVBV rates in native palo verde species and two palo verde hybrids sampled here. Detection of asymptomatic PVBV infection, despite relative low frequencies in some instances, may suggest a requirement for a latent period between time of inoculation, virus multiplication, and systemic infection. The influence of one or more of these or other variables could suggest seasonally-dependent systemic movement and/or uneven virus distribution in different branches or other tree parts, possibly influenced by timing and extent of mite colonization that leads to virus transmission (Martin et al., 2021; Takahashi et al., 2019).

Eriophyid mite are suspect or have been established as the arthropod vector of emaravirus species, and they are host-specific as are their counterpart emaravirus (Buzkan et al., 2019; Jensen, 1996; Kubota et al., 2020). Further, field populations of other eriophyid mites have been reported to be 17 times denser on symptomatic plants and/or tissues, and virus transmission was more efficient by mites colonizing rapidly growing plant parts (Epstein and Hill, 1999). In turn, those plant parts were more susceptible to mite multiplication and virus infection. Studies have shown that mites can cause direct and indirect damage to epidermal cells on which they feed, and that feeding damage can result in cell collapse and accumulation of lignin-like compounds in the walls of nearby cells. Indirectly, certain herbivorous mites appear to pre-condition the host by injecting salivary secretions that disrupt host defenses that result in increased jasmonic acid (JA) precursors (Villarroel et al., 2016), whereas spider mite feeding resulted in suppression of JA downstream, independent of salicylic acid accumulation (JA agonist) (see references in: de Lillo et al., 2018).

The effects of the virus-infected plants on the biology of the eriophyid mites infesting them are poorly known, however, the co-evolutionary advantages are thought to be beneficial both to the virus and the mite vector. The mite species, *Aceria tosichella* and *A. cajani*, has been shown to experience increased fecundity and density, respectively, when colonizing HPWMoV-infected wheat plants, and pigeonpea sterility mosaic virus (PPSMV)-infected pigeon pea plants, respectively (Jones et al., 2004; Kulkarni et al., 2002; Latha and Doraiswamy, 2008; Murugan et al., 2011; Reddy and Nene, 1980; Siriwetwiwat, 2006; Skoracka et al., 2013). Consistent with previously reported eriophyid mite-emaravirus study systems, most broom-symptomatic palo verde trees supported significant populations of the palo verde eriophyid mite. Interestingly, the two palo verde hybrids were asymptomatic but positive for PVBV-infection and harbored heavy mite infestations. Efforts to demonstrate mite-mediated PVBV transmission were hindered in this study (unpublished results) due to the difficulty finding mites on PVBV-free trees, which were colonized by few if any palo verde mites. Although more studies are needed to explore the biological basis for these observations, the high numbers of mites present on branches and leaflets of mite-infested PVBV-infected palo verde tree species and hybrids (Table 3) point to a complex virus-vector relationship, analogous to those reported for well-studied emaravirus-mite complexes (De Lillo et al., 2018). Finally, preliminary experiments showed that PVBV was detectable in cohorts of mites collected from symptomatic trees by RT-PCR amplification of the NP gene (authors, unpublished; data not shown), which is consistent with other reports that emaraviruses are detectable in RNA purified from suspect eriophyid mite vectors (Caglayan et al., 2012; Druciarek et al., 2019).

Here, nineteen complete genomes of PVBV, each consisting of RNA segments 1–5, were *de novo* assembled following Illumina® sequencing of four species and two hybrid palo verde. Among them are the five isolates for which the PVBV RNA1–4 segments have been previously report (Ilyas et al., 2018). Consistent with previously characterized genomes of emaraviruses classified in the family *Fimoviridae* (Digiaro et al., 2024), the five PVBV RNA genome segments contained a virus-specific, highly conserved, complementary sequence located on the 5′ and 3′ terminus of each RNA segment. This canonical sequence was identified on all *de novo* assembled, near full-length genome segments and on the RNA segments 1–5 that were PCR-amplified from the PVBV field isolates recovered. Also, the RNA1–4 sequences determined in this study were indistinguishable from those previously reported (Ilyas et al., 2018). Two full-length genomes (denoted A and B) were identified in Mexican and blue palo verde isolates 23.27–1A/B, and 23.27–4A/B. This is the first evidence of an emaravirus specie exhibiting two divergent genome variants within the same host. This unique observation of two complete sets of RNA1–5 segments indicates mixed infection of two different isolates in a palo verde tree, suggests a probable virus adaptational strategy, potentially enhancing functional diversity, host range expansion, and evolutionary flexibility. Also, two divergent RNA1 segments were recovered from isolate 21.131–1, while two divergent RNA2 segments were identified for isolate 21.101–6, reminiscent of HPWMoV for which two divergent RNA3 genome segments were reported from the same plant (Tatineni et al., 2014). Similar observations have also been reported for other emaravirus species (Buzkan et al., 2019; Kubota et al., 2021, 2020). These results were consistent among the PVBV genomes recovered by *de novo* assembly (RNAseq) and genomes amplified by RT-PCR from the above samples and subjected to confirmatory sequencing.

High mutation rates, which are characteristic among other RNA viruses (Domingo et al., 2021), was observed among the genomes of PVBV isolates reported here. This is consistent with the quasispecies nature of other RNA viruses that have been shown to have a propensity for adaptation through increased or decreased fitness (Domingo et al., 2021). The high mutation rates observed for PVBV genomes may possibly be explained by the adaptation to multiple palo verde species, feasibly through *Multiple species* jumps. Until this study, the only known host of PVBV was the blue palo verde. However, these results indicate that the PVBV host range spans at least four palo verde species, including two palo verde hybrids.

Among the PVBV isolates, pairwise distance analysis of the five PVBV coding regions (amino acids) indicated that the intra-isolate variation ranged from minimal to relatively extensive. In general, virus-encoded non-structural proteins exhibited higher amino acid dissimilarities than the structural proteins, at 73–100 %. Among the five PVBV ORFs, the amino acid identity of the PVBV RNA5-encoded protein was the most variable, an observation that is consistent among all known emaraviruses. Although intra-specific amino acid sequence similarities among emaravirus RNA genome segments (coding regions) can be highly variable (Katsiani et al., 2020; Patil et al., 2017). Interestingly, the PVBV RNA3 (NP coding region) segment of PVBV shared its’ highest identity with raspberry leaf blotch virus (RLBV), taxonomically classified as *Emaravirus idaeobati* NP ORF (Dong et al., 2016), the other PVBV genomic segments diverged substantially from the rest of the RLBV genome. The PVBV GP and the NP, which encode structural proteins, were highly conserved at the nucleotide and amino acid levels and shared the greatest amino acid identity at 93–100 %, respectively. This is consistent with knowledge that even a few mutations in the GP and/or NP gene(s), which are implicated in eriophyid mite vector-mediated transmission, genome (RNA) encapsidation, and envelope formation, could undermine essential virus functions (Domingo, 2002; Sanjuán and Domingo-Calap, 2016).

The Bayesian phylogeny for the concatenated and individual RNA segments of the genome showed that PVBV isolates cluster into distinct clades with statistically significant posterior probability support, providing evidence for PVBV diversification in its’ palo verde hosts. The concatenated RNA1–5 segments (complete genome) formed two well-resolved PVBV clades. Clade I contained most PVBV isolates that grouped with diverse sub-clades, whereas the clade II isolates were represented by two divergent PVBV variants. Interestingly, the HPWMoV lineages L1 and L2, from Australia were similarly resolved by phylogenetic analysis (Jones et al., 2023). Within the PVBV clade I, three well-supported sub-clades containing PVBV isolates represented all six palo verde species, while subclade Ib contained PVBV isolates from a single BPV tree. This pattern suggests that the PVBV isolates sequenced in this study are closely related. By comparison, a Bayesian analysis of the NP gene sequence of fig mosaic virus isolates (Walia et al. 2014) from California resolved three main clades. Although the PVBV genomes / isolates did not cluster with a basis in host species or geographically-distinct collection sites, the clusters may indicate divergence potentially associated with palo verde genetic lineages, including those resulting from intraspecific hybridization, which is known to occur among palo verde. Nonetheless, all are apparently transmitted by one mite species that is apparently host-specific to *Parkinsonia* spp. Even so, PVBV-host specificity appears to be less important than maintenance of putative conserved eriophyid mite vector transmission determinants. Although isolates of blackberry leaf mottle-associated virus (BLMaV), taxonomically classified as *Emaravirus rubi* showed geographical structuring by certain populations (Hassan and Tzanetakis, 2019), this could be explained by genetic bottlenecks, feasibly to give rise to isolated populations. To date, similar structuring was recently reported for perilla mosaic virus (PerMV), taxonomically classified as *Emaravirus perillae*, but has not been observed for other emaraviruses (Dong et al., 2016; Katsiani et al., 2020; Verchot et al., 2023; Walia et al., 2014), however, such patterns could be obfuscated among emaravirus populations that infect vegetatively propagate plant hosts, due to long-distance transport of the virus-host combination and subsequent isolation.

Overall, the PVBV isolates exhibit a range of genomic variability. The most divergent isolates were recovered from trees supporting mixed PVBV infections. Also, several predicted PVBV recombinants were detected in single and mixed infections regardless of whether the individual or concatenated RNA1–5 segments of the PVBV genomes were analyzed by RDP (*P* < 0.05) and the PHI test. To some extent, intraspecific recombination appears important to PVBV diversification. In particular, recombination predicted by the concatenated RNA-1 analysis involving isolate 23.27–2A, for which RNA1 of 23.27–2B was identified as the major parent, was consistent with hyper-recombination scenarios previously reported for emaraviruses found to harbor two copies of the same RNA genome segment. Among them are a predicted recombinants of BLMaV that involved the MP gene (Hassan and Tzanetakis, 2019) and recombinants with multiple viral RNA segments e.g. RNA 1, 2, 4, and 5, of cajani syn. pigeonpea sterility mosaic virus 1 (PPSMV-1). The notable divergence among PVBV isolates 23.27–1B and 23.27–13 is suggestive of genetic drift, which may possibly reflect enhanced fitness and/or lineage separation. Such outcomes could possibly be explained by adaptation to interspecific hybridization between palo verde species, mite-virus interactions, or environmental stress, which are all within the realm of the quasi-species nature of RNA viruses that provides inherent capacity to undergo rapid population shifts (Domingo, 2002).

The diversity among PVBV genomes is relatively extensive, based on nucleotide diversity values spanning 0.0221 to 0.0846 and on the high haplotype diversity index (Hd). The dN-dS and codon selection tests identified negative selection as the predominant force acting on the PVBV coding regions. Notably, RNA3 had the lowest ratio, at 0.0256, which probably reflects functional constraints of the nucleocapsid gene (NP, 35 kDa) given that it is essential for encapsidation and presumably, mite transmission. Analogously, the FMV RNA3 and RNA4 genomic segments were shown to exhibit the lowest dN/dS ratio among the five FMV viral RNAs, respectively (Walia et al., 2014). Positive selection was detected acting on a limited number of sites for the GP, MP, and RdRp proteins, all that are expected to be essential for the infection cycle in the host plant, namely virus replication, systemic infection, and encapsidation. The results of the neutrality tests, Tajima’s *D* and Fu, and Li’s D* and F*, predicted the prevalence of low-frequency genetic variants, possibly reflective of population expansion or recent host adaptation. The FEL, MEME, FUBAR, and SLAC programs consistently identified codons that have undergone negative selection, while MEME and FUBAR tests identified sites in the viral GP and MP undergoing positive selection. Although speculative, positive selection may derive from virus-host interactions, namely, those involved in adaptation necessary to counter host defense response, feasibly shared in common by the different palo verde species.

The increased prevalence of PVBV in *Parkinsonia* spp. trees during the last 20 years in southern Arizona, and the first consistent associations of PVBV infection of palo verde trees with witches’ broom symptoms are indicative of a dynamic pathosystem and of potential ecological implications in Arizona desert ecosystems. The consistent association of palo verde eriophyid mites with PVBV-infected trees of all four species, and only low-level to no mite infestations on most asymptomatic trees, can serve as diagnostic indicators of suspect virus infections. These criteria cannot be used as reliable indicators of virus infection of the two palo verde hybrids, which are asymptomatic, and may or may not be PVBV-infected, but may harbor mites.

The restricted host range of the palo verde eriophyid mite and its tight association with the leaflets and developing flower buds points to an organ-associated tropism with *Parkinsonia* spp., This scenario may suggest an epidemiological relationship between mite-flower bud association and PVBV seed-transmission (preliminary results). Also, eriophyid mites have been observed feeding near and on plant trichomes where they are most dense, suggesting the host provides a protected environment for both the mites and eggs. Finally, eriophyid mites have been reported to stimulate the growth of abnormal trichomes for such purposes (De Lillo et al., 2018). Thus, the relationship between the mites and palo verde plant trichomes, which appeared altered on mite-colonized leaflets and buds (Fig. 1A), is of great interest to understanding the basis for the development of witches’ broom phenotype, which is possibly caused by both mite feeding and the virus infection. The high populations of *A. cercidii* observed on virus-infected trees, compared to small population sizes or few mites on virus-free trees suggest that palo verde mite may be attracted to PVBV-infected trees and/or that mite reproduction is enhanced in virus-infected trees (see references in: De Lillo et al., 2018).

Studies are now needed to evaluate the relationship between PVBV prevalence and eriophyid mite infestations in *Parkinsonia* spp. and identify the environmental factors that contribute to vector dispersal and virus transmission. Emaraviruses encode a glycoprotein, which strongly suggests they may infect and therefore replicate in eriophyid mite vector cells. Definitive studies are needed to test this hypothesis and to identify the viral-encoded determinant(s) and concomitant virus receptors that interact to facilitate PVBV transmission.

## Ethics approval and consent to participate

Not applicable.

## Consent for publication

Not applicable.

## Data availability

Sequence data supporting conclusions in this manuscript are available in the NCBI GenBank database as the Accession numbers PV943489-PV943616. Other information supporting the conclusions in this article are included as supplementary file(s).

## Funding

This study was supported by Arizona Department of Agriculture Specialty Crop Block Grants: SCBGP20-18 and SCBGP22-41 awarded to UKS and JKB School of Plant Sciences, University of Arizona, Tucson, Arizona, 85721 USA.

## CRediT authorship contribution statement

**Raphael O. Adegbola:** Writing – original draft, Visualization, Validation, Software, Methodology, Investigation, Formal analysis, Data curation, Conceptualization. **Dinusha C. Maheepala:** Writing – original draft, Validation, Software, Methodology, Formal analysis, Data curation. **Ursula K. Schuch:** Writing – review & editing, Writing – original draft, Supervision, Resources, Project administration, Methodology, Funding acquisition, Formal analysis, Data curation, Conceptualization. **Judith K. Brown:** Writing – review & editing, Writing – original draft, Supervision, Resources, Project administration, Methodology, Investigation, Funding acquisition, Conceptualization.

## Declaration of competing interest

The authors declare they have no competing interests.

## Data Availability

Data will be made available on request.

## Appendix Supplementary materials

Image, application 1

Image, application 2

Image, image 3

Image, image 4

Image, image 5

Image, image 6

Image, image 7
