# Supplementary material for: Prevalence, host range, and characterization of multiple Palo verde broom emaravirus genomes and eriophyid mites from Parkinsonia spp. in Arizona
Source: Virus Res. 2025 Oct 16;361:199643. doi: 10.1016/j.virusres.2025.199643 (PMC12596537; doi:10.1016/j.virusres.2025.199643)
Supplement: Supplementary file 1 [file mmc1.docx]

**Supplementary Table 2A.** Summary of PVBV infection, witches’ broom symptoms, and presence of mites across all species.

| PVBV infection | Eriophyid mites | Witches broom symptoms | Plants *  (No.) | BPV | MPV | FPV | SPV | HDMPV | HPV |
| --- | --- | --- | --- | --- | --- | --- | --- | --- | --- |
| + | + | + | 11 (21%) | 8 | 3 | - | - | - | - |
| + | + | - | 16 (30%) | 1 | 5 | 1 | 1 | 5 | 3 |
| + | - | + | 8 (15%) | 5 | 1 | 2 | - | - | - |
| + | - | - | 8 (15%) | 2 | - | 2 | 2 | 1 | - |
| - | + | + | 1 (2%) | 1 | - | - | - | - | - |
| - | + | - | 6 (12%) | 1 | - | 2 | 1 | 2 | - |
| - | - | + | 2 (4%) | - | - | 2 | - | - | - |
| - | - | - | 2 (4%) | - | - | - | 2 | - | - |

*Out of the 70 plants samples, 53 had observations for each of the three variables.

BPV = blue palo verde, MPV = Mexican palo verde, FPV = Foothills palo verde, SPV = Sonoran palo verde, HDMPV = Hybrid Desert Museum palo verde, HPV = Hybrid palo verde

**Supplementary Table 2B.** Summary of PVBV infection and witches’ broom symptoms averaged over all plants with observations for those two variables.

| PVBV infection | Witches broom symptoms | Plants *  (No.) | BPV | MPV | FPV | SPV | HDMPV | HPV |
| --- | --- | --- | --- | --- | --- | --- | --- | --- |
| + | + | 34 (50%) | 18 | 9 | 7 | - | - | - |
| + | - | 22 (34%) | 3 | 5 | 2 | 3 | 6 | 3 |
| - | + | 4 (6%) | 1 | - | 3 | - | - | - |
| - | - | 7 (10%) | 1 | - | 2 | 3 | 1 | - |

*Out of the 70 plants, 67 plants had observations for the two variables.

BPV = blue palo verde, MPV = Mexican palo verde, FPV = Foothills palo verde, SPV = Sonoran palo verde, HDMPV = Hybrid Desert Museum palo verde, HPV = Hybrid palo verde

**Supplementary Table 2C.** Summary of PVBV infection and the presence of mites averaged over all plants with observations for those two variables.

| PVBV infection | Eriophyid mites | Plants *  (No.) | BPV | MPV | FPV | SPV | HDMPV | HPV |
| --- | --- | --- | --- | --- | --- | --- | --- | --- |
| + | + | 27 (50%) | 9 | 8 | 1 | 1 | 5 | 3 |
| + | + | 15 (29%) | 7 | 1 | 4 | 2 | 1 | - |
| - | + | 7 (13%) | 2 | - | 2 | 1 | 2 | - |
| - | + | 4 (8%) | - | - | 2 | 2 | - | - |

*Out of the 70 plants, 54 plants had observations for the two variables.

BPV = blue palo verde, MPV = Mexican palo verde, FPV = Foothills palo verde, SPV = Sonoran palo verde, HDMPV = Hybrid Desert Museum palo verde, HPV = Hybrid palo verde

**Supplementary Table 2D.** Summary of witches’ broom symptoms and presence of mites averaged over all plants with observations for those two variables.

| Witches broom symptoms | Eriophyid mites | Plants *  (No.) | BPV | MPV | FPV | SPV | HDMPV | HPV |
| --- | --- | --- | --- | --- | --- | --- | --- | --- |
| + | + | 11 (22%) | 8 | 3 | - | - | - | - |
| + | - | 9 (18%) | 5 | 1 | 3 | - | - | - |
| - | + | 21 (44%) | 2 | 5 | 3 | 2 | 6 | 3 |
| - | - | 8 (16%) | 2 | - | 1 | 4 | 1 | - |

*Out of the 70 plants, 49 plants had observations for the two variables.

BPV = blue palo verde, MPV = Mexican palo verde, FPV = Foothills palo verde, SPV = Sonoran palo verde, HDMPV = Hybrid Desert Museum palo verde, HPV = Hybrid palo verde

**Supplementary Table 3.** Sites at which positive and negative selection were detected, and non-synonymous vs. synonymous substitution rates (dN/dS) for the encoded genes of palo verde broom virus (PVBV) from different *Parkinsonia* spp.

| Gene | FEL* | | MEME | FUBAR | | SLAC (dN/dS) | | |
| --- | --- | --- | --- | --- | --- | --- | --- | --- |
|  | Negative** | Positive | Positive | Positive | Negative | Positive | Negative |  |
| RdRp | 969 | 0 | 3 | 2 | 1891 | 0 | 340 |  |
| GP | 53 | 0 | 4 | 0 | 163 | 0 | 0 |  |
| NP | 14 | 0 | 0 | 1 | 23 | 0 | 3 |  |
| MP | 99 | 0 | 1 | 0 | 159 | 0 | 10 |  |
| HP | 39 | 0 | 0 | 0 | 41 | 0 | 3 |  |

*FEL (Fixed Effects Likelihood), MEME (Mixed Effects Model of Evolution), FUBAR (A Fast, Unconstrained Bayesian AppRoximation for Inferring Selection), SLAC (Single Likelihood Ancestor Counting)

**negative selection pressure and positive selection pressure

#ratio of non-synonymous (*dN*) and synonymous (*dS*) sites


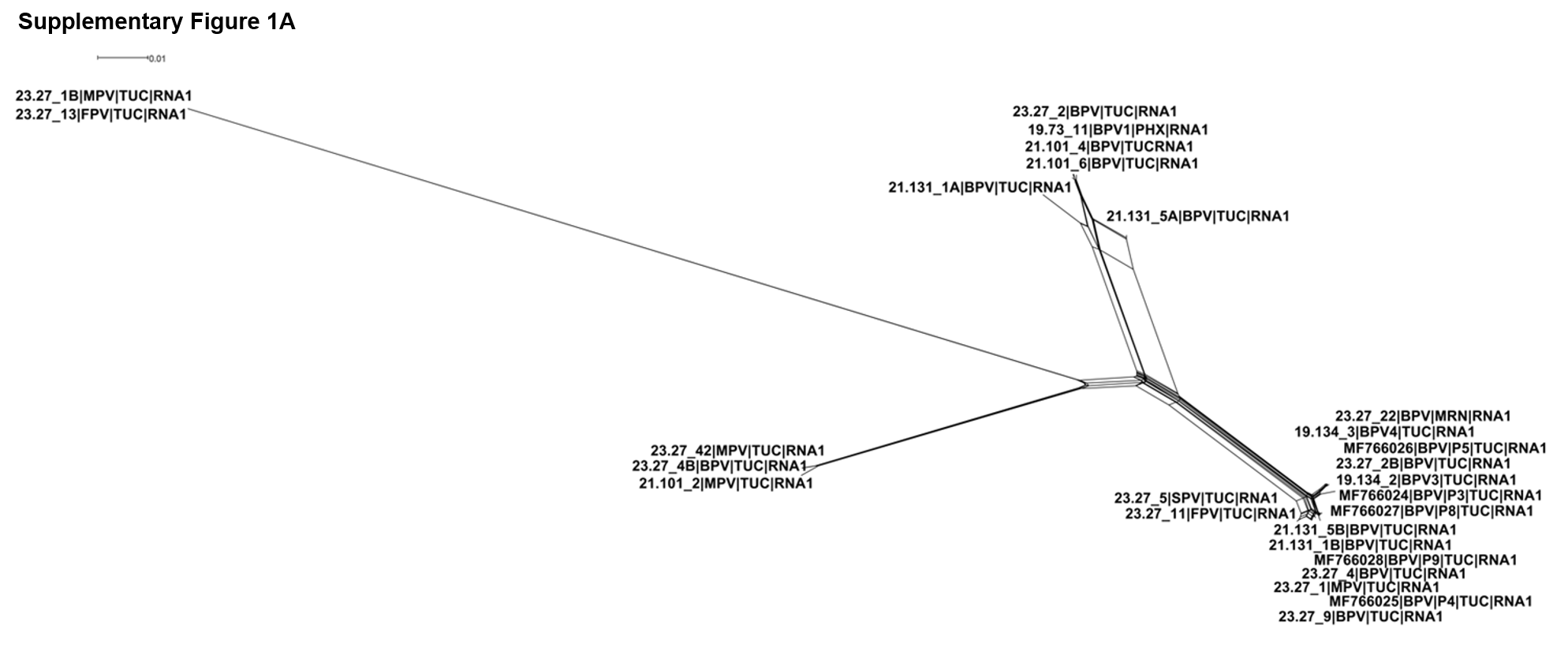


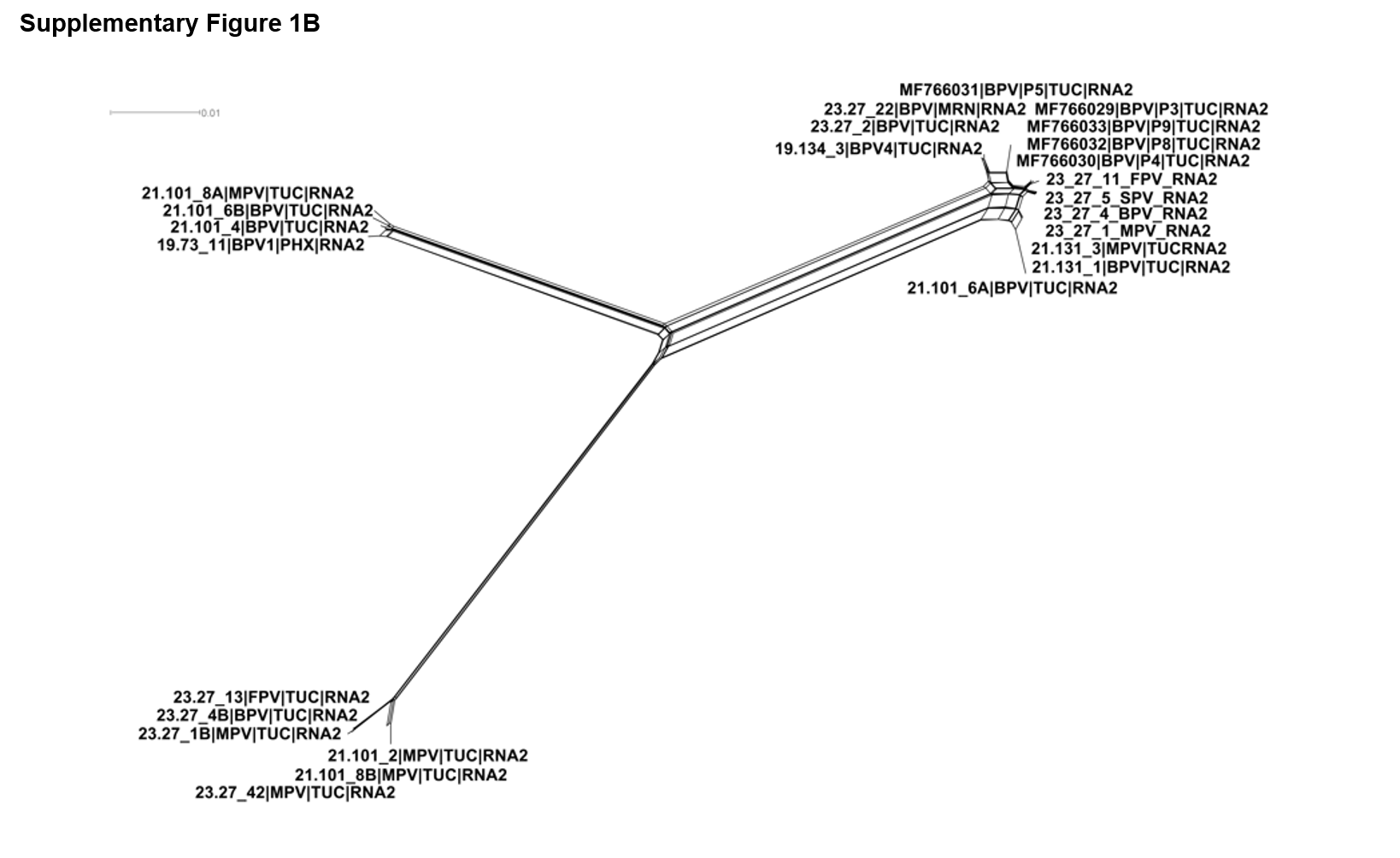


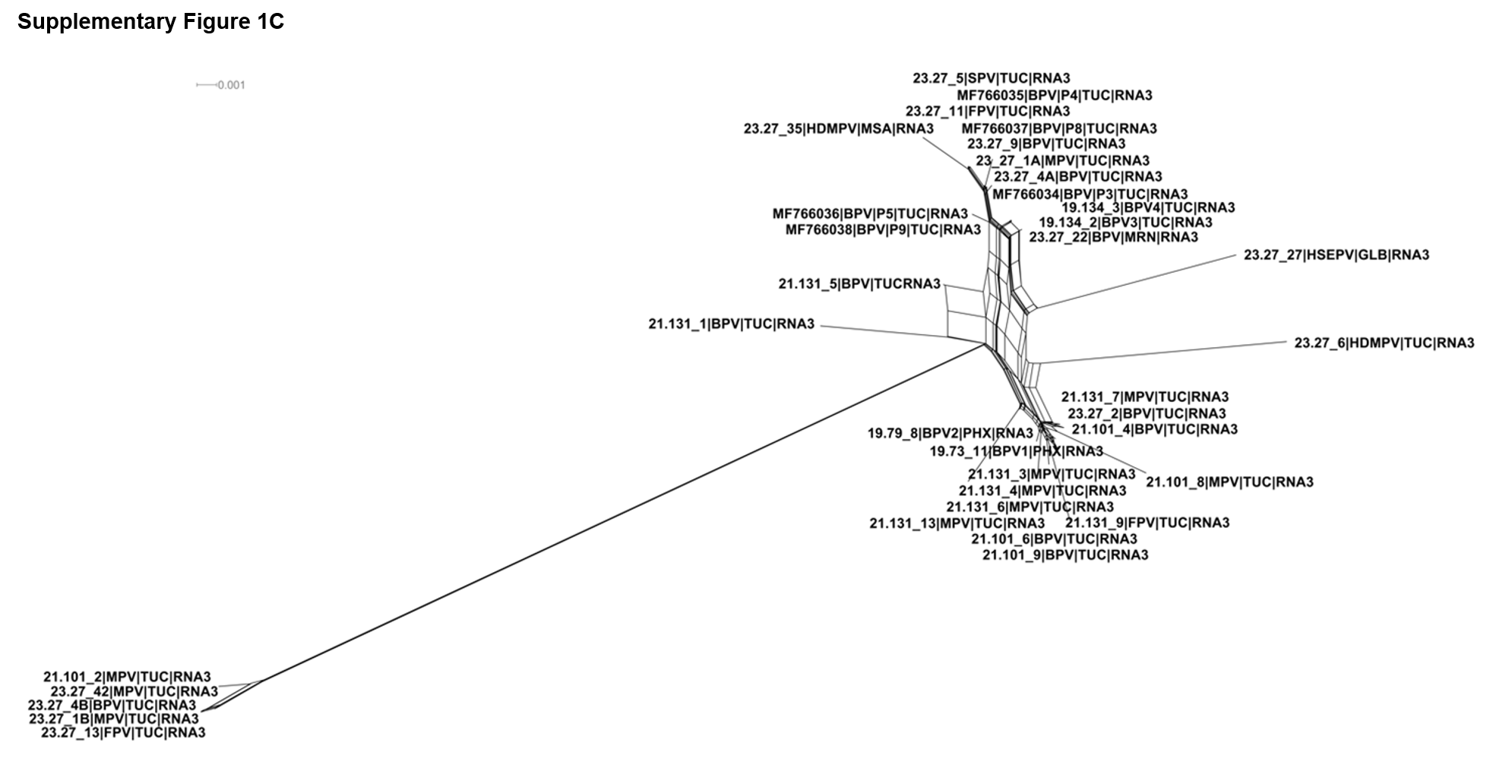


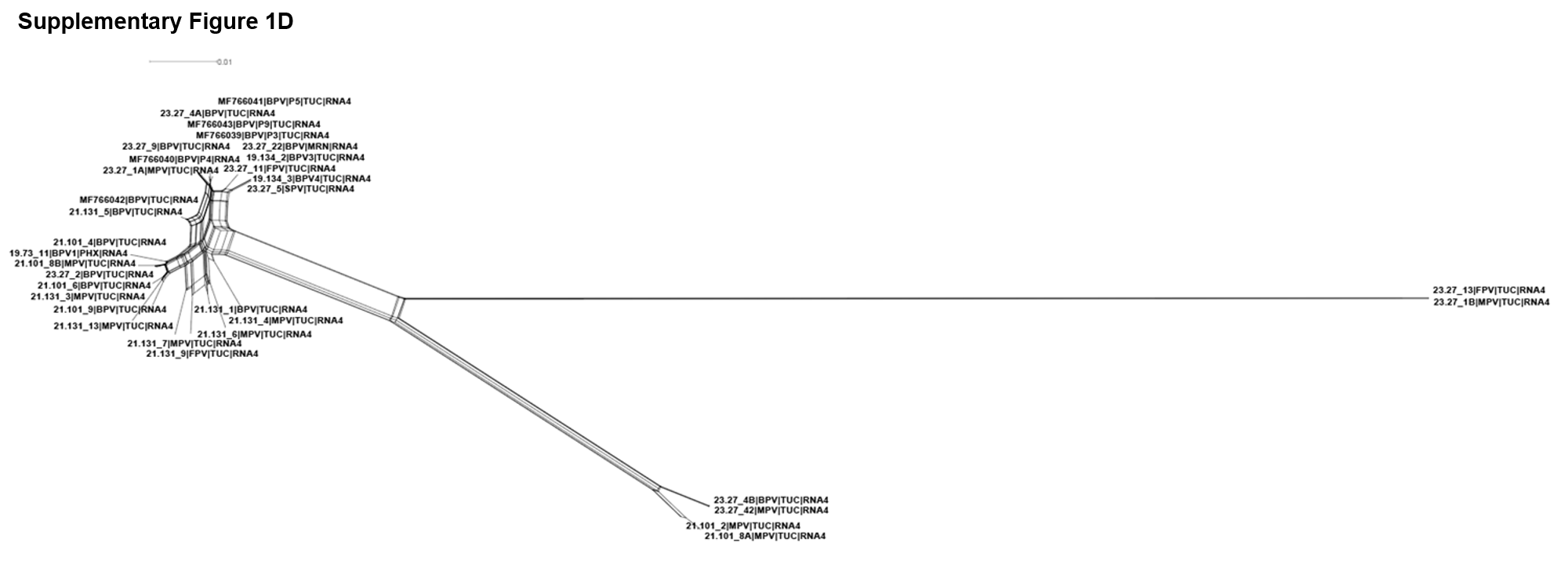


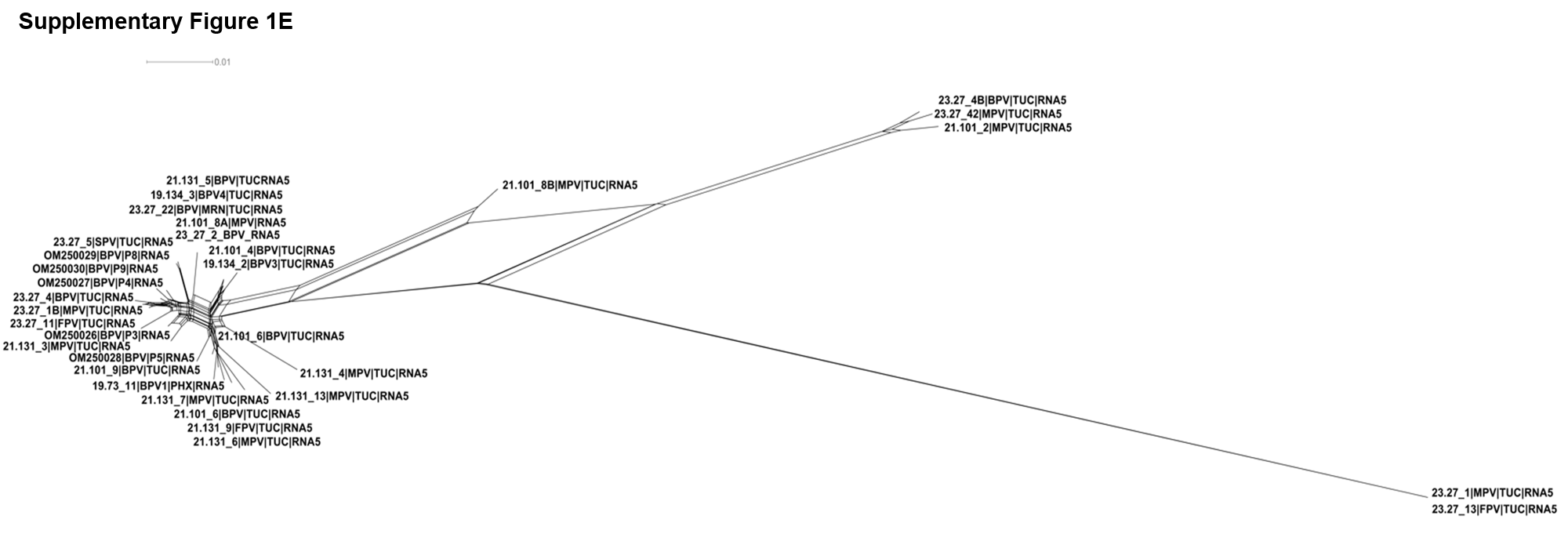


**Figure S1A-E.** Phylogenetic network analysis of palo verde broom virus (PVBV) individual genomic RNA segments of isolates characterized in this study and reference isolates sequences available in the GenBank database. The analysis was carried out using the NeighborNet algorithm implemented in SplitsTree4 v4.14.6 (Hudson and Bryant, 2005). The tree was reconstructed using Uncorrected_P method, and the network was drawn using EqualAngle. The bifurcating paths indicate a low probability of recombination. The host species abbreviations are: blue palo verde (BPV), foothills palo verde (FPV), Mexican palo verde (MPV), Sonoran palo verde (SPV) geographic locations are abbreviated as follows: Alabama (AL), Argentina (AR), Brazil (BR), China (CN), Florida (FL), Georgia (GA), Louisiana (LA), North Carolina (NC), Oklahoma (OK), South Carolina (SC), South Korea (KR), and Texas (TX).
