# Supplementary figures and images for: Prevalence, host range, and characterization of multiple Palo verde broom emaravirus genomes and eriophyid mites from Parkinsonia spp. in Arizona
Source: Virus Res. 2025 Oct 16;361:199643. doi: 10.1016/j.virusres.2025.199643 (PMC12596537; doi:10.1016/j.virusres.2025.199643)

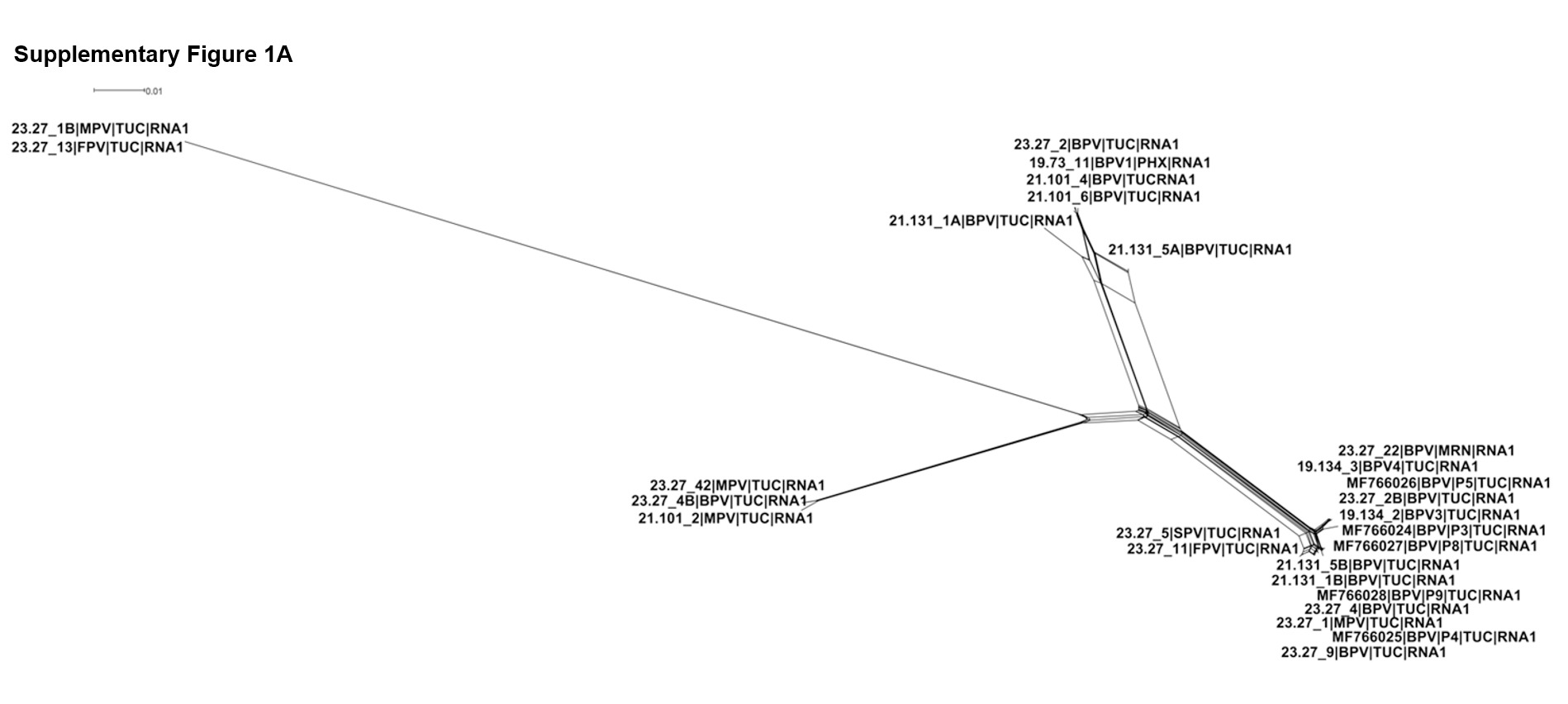

Supplement: Supplementary file 3 [file mmc3.jpg]

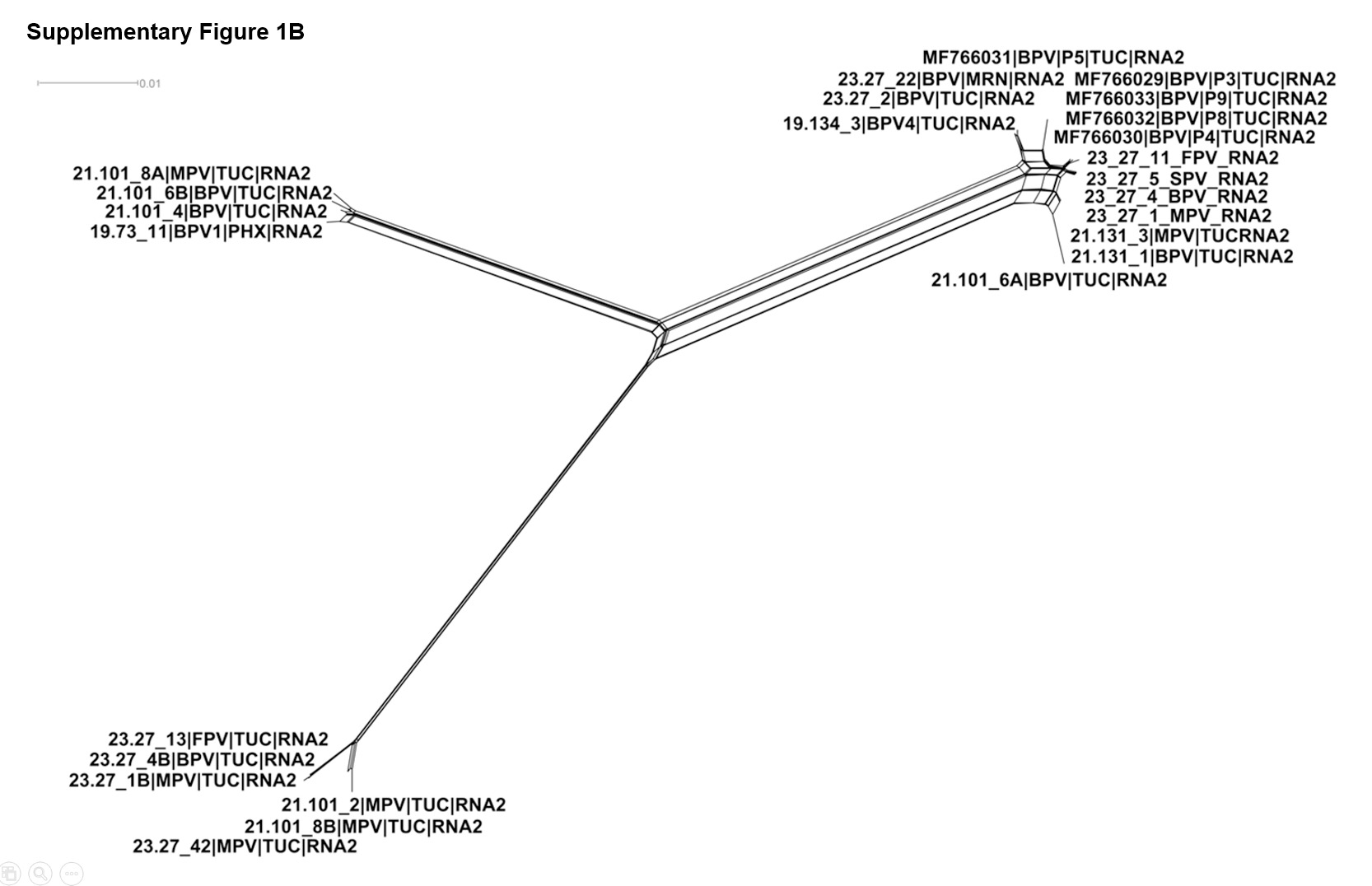

Supplement: Supplementary file 4 [file mmc4.jpg]

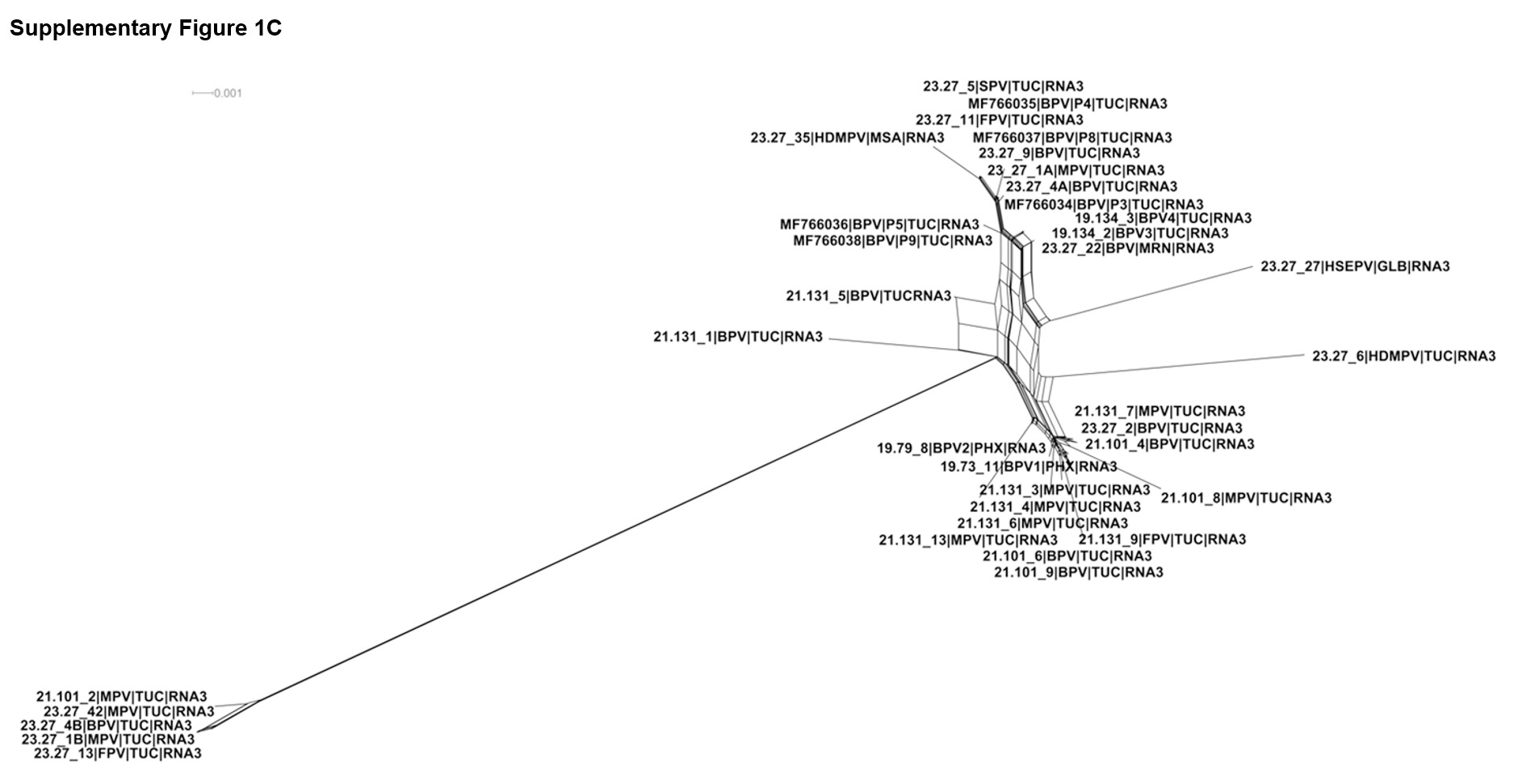

Supplement: Supplementary file 5 [file mmc5.jpg]

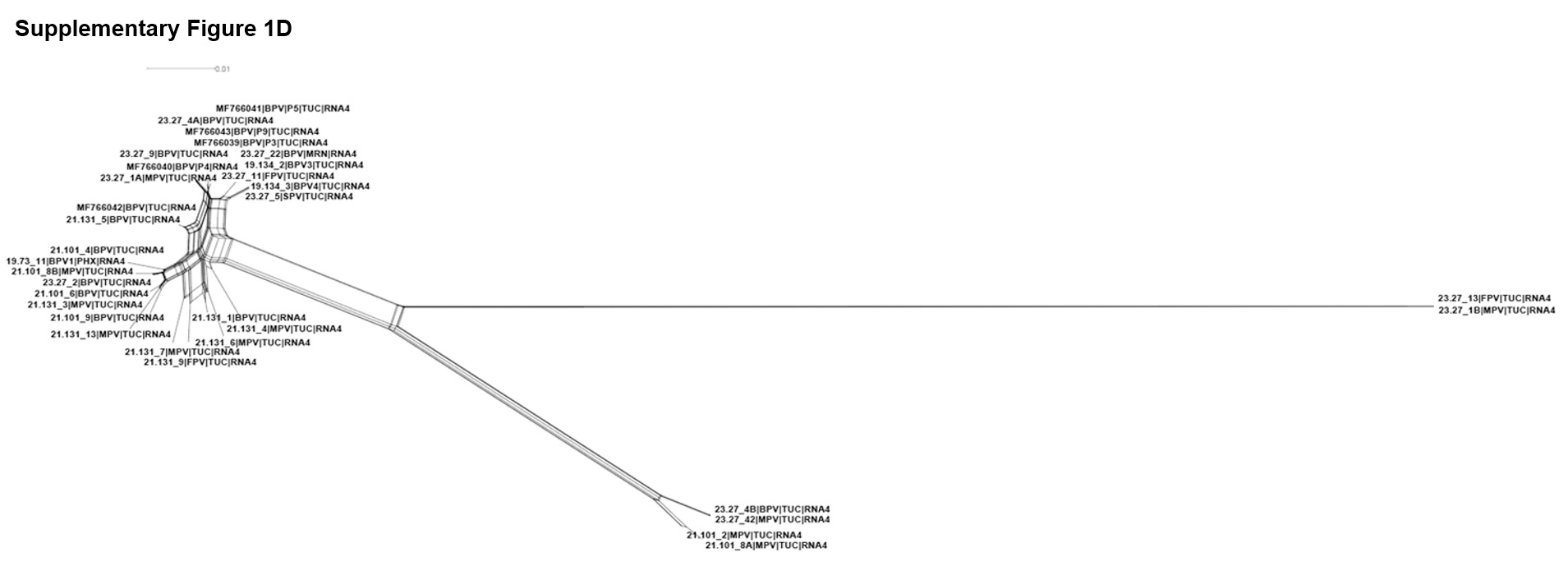

Supplement: Supplementary file 6 [file mmc6.jpg]

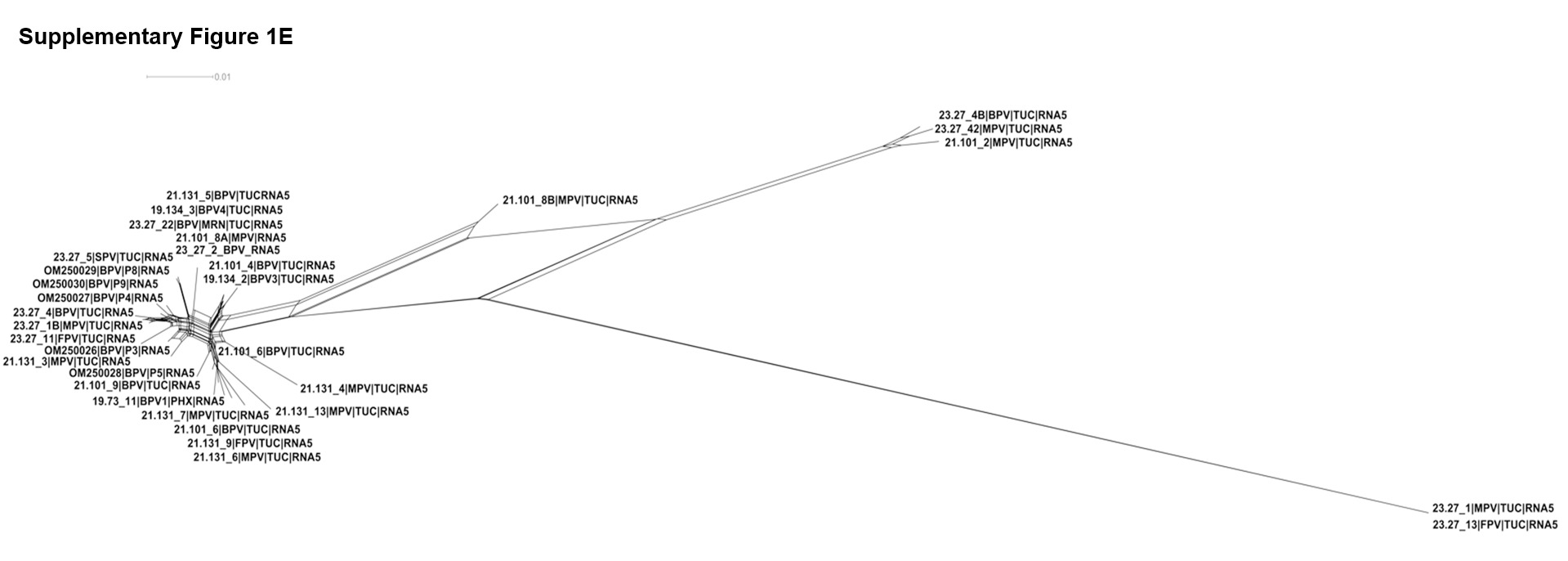

Supplement: Supplementary file 7 [file mmc7.jpg]
